# Pinosylvin: A Multifunctional Stilbenoid with Antimicrobial, Antioxidant, and Anti-Inflammatory Potential

**DOI:** 10.3390/cimb47030204

**Published:** 2025-03-18

**Authors:** Argyrios Periferakis, Aristodemos-Theodoros Periferakis, Lamprini Troumpata, Konstantinos Periferakis, Spyrangelos Georgatos-Garcia, Georgia Touriki, Christiana Diana Maria Dragosloveanu, Ana Caruntu, Ilinca Savulescu-Fiedler, Serban Dragosloveanu, Andreea-Elena Scheau, Ioana Anca Badarau, Constantin Caruntu, Cristian Scheau

**Affiliations:** 1 Department of Physiology, The “Carol Davila” University of Medicine and Pharmacy, 050474 Bucharest, Romania; 2 Akadimia of Ancient Greek and Traditional Chinese Medicine, 16675 Athens, Greece; 3 Elkyda, Research & Education Centre of Charismatheia, 17675 Athens, Greece; 4 Pan-Hellenic Organization of Educational Programs (P.O.E.P.), 17236 Athens, Greece; 5 Tilburg Institute for Law, Technology, and Society (TILT), Tilburg University, 5037 DE Tilburg, The Netherlands; 6 Corvers Greece IKE, 15124 Athens, Greece; 7 Faculty of Law, Democritus University of Thrace, 69100 Komotini, Greece; 8 Department of Ophthalmology, Faculty of Dentistry, The “Carol Davila” University of Medicine and Pharmacy, 020021 Bucharest, Romania; 9 Department of Ophthalmology, Clinical Hospital for Ophthalmological Emergencies, 010464 Bucharest, Romania; 10 Department of Oral and Maxillofacial Surgery, “Carol Davila” Central Military Emergency Hospital, 010825 Bucharest, Romania; 11 Department of Oral and Maxillofacial Surgery, Faculty of Dental Medicine, Titu Maiorescu University, 031593 Bucharest, Romania; 12 Department of Internal Medicine, The “Carol Davila” University of Medicine and Pharmacy, 050474 Bucharest, Romania; 13 Department of Internal Medicine and Cardiology, Coltea Clinical Hospital, 030167 Bucharest, Romania; 14 Department of Orthopaedics and Traumatology, The “Carol Davila” University of Medicine and Pharmacy, 050474 Bucharest, Romania; 15 Department of Orthopaedics, “Foisor” Clinical Hospital of Orthopaedics, Traumatology and Osteoarticular TB, 021382 Bucharest, Romania; 16 Department of Radiology and Medical Imaging, “Foisor” Clinical Hospital of Orthopaedics, Traumatology and Osteoarticular TB, 021382 Bucharest, Romania; 17 Department of Dermatology, “Prof. N.C. Paulescu” National Institute of Diabetes, Nutrition and Metabolic Diseases, 011233 Bucharest, Romania

**Keywords:** pinosylvin, antimicrobial effects, antioxidant, anti-inflammatory, anticancer, ethnomedicine, pathophysiology

## Abstract

Stilbenoids are a category of plant compounds exhibiting notable health-related benefits. After resveratrol, perhaps the most well-known stilbenoid is pinosylvin, a major phytochemical constituent of most plants characterised by the pine spines among others. Pinosylvin and its derivatives have been found to exert potent antibacterial and antifungal effects, while their antiparasitic and antiviral properties are still a subject of ongoing research. The antioxidant properties of pinosylvin are mostly based on its scavenging of free radicals, inhibition of iNOS and protein kinase C, and promotion of HO-1 expression. Its anti-inflammatory properties are based on a variety of mechanisms, such as COX-2 inhibition, NF-κB and TRPA1 activation inhibition, and reduction in IL-6 levels. Its anticancer properties are partly associated with its antioxidant and anti-inflammatory potential, although a number of other mechanisms are described, such as apoptosis induction and matrix metalloproteinase inhibition. A couple of experiments have also suggested a neuroprotective potential. A multitude of ethnomedical and ethnobotanical effects of pinosylvin-containing plants are reported, like antimicrobial, antioxidant, anti-inflammatory, hepatoprotective, and prokinetic actions; many of these are corroborated by recent research. The advent of novel methods of artificial pinosylvin synthesis may facilitate its mass production and adoption as a medical compound. Finally, pinosylvin may be a tool in promoting environmentally friendly pesticide and insecticide policies and be used in land remediation schemes.

## 1. Introduction

In current times, there is a wide availability of pharmaceutical agents for treating the causes and symptoms of a broad spectrum of pathologies [1,2,3]. However, many of these drugs, albeit effective, are associated with side-effects, which may be comparatively mild or even severe [4,5,6,7,8,9,10]. In the specific case of antimicrobial agents, microbial resistance has become another pronounced problem [11,12,13,14].

In an effort to find alternative therapeutical agents and based also on a rich medical–ethnobotanical tradition of different cultures, phytomedicine has been steadily gaining ground in the last decades [15,16,17,18,19,20,21,22,23,24,25,26,27,28]. Numerous compounds have been explored for their potential medicinal effects such as capsaicin, quercetin, curcumin, and kaempferol, among others [29,30,31,32,33,34,35,36,37,38,39].

Pinosylvin, a stilbenoid polyphenol mostly found in plants of the Pinaceae family, is the focus of this paper. It is believed that pinosylvin and its derivatives form part of a defence mechanism of the plants producing them, against microorganisms and insects [40]; it was isolated and identified for the first time in 1939 [41]. Currently, there exists a wealth of data on the antibacterial, antifungal, antiparasitic, antiviral, antioxidative, anti-inflammatory, anti-carcinogenic, and neuroprotective properties and potential.

In this review, we will provide a comprehensive overview of the current knowledge available on pinosylvin and its derivatives, presenting the research pertaining to its aforementioned activities and potential, in a clear and detailed manner. The associated mechanisms and potential links between the antioxidant and anti-inflammatory applications will also be explored, as well as the available evidence for its anticancer properties and main traditional uses of pinosylvin-containing plants. It is our intention, via this mode of presentation, and based on our detailed bibliographical research, using Google Scholar, PubMed, and Scopus databases, to incorporate the current state of knowledge on this topic and to provide directions for future research.

## 2. Biosynthesis and Bioavailability of Pinosylvin

**Figure 1:**
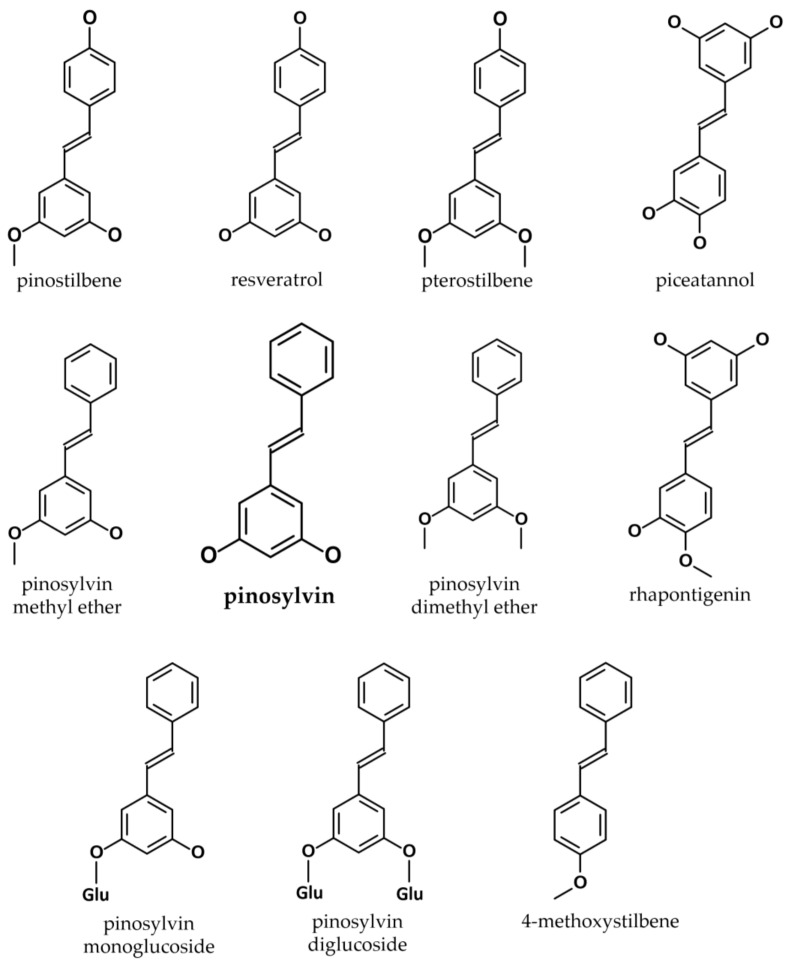
Pinosylvin and its most common derivatives. Glu = glucose.

In general, all stilbenoids are phenolic derivatives of the phenylpropanoid pathway [42]. Their common structural characteristic is the stilbene core of phenyl rings connected by a methylene bridge [43]; based on the precise arrangement of these phenyl rings and a variety of substitution patterns, stilbenoids are classified in various subclasses [44]. The stilbenoids are hydroxylated stilbene derivatives, having a biosynthesis pathway which shares many steps with that of chalcones [45,46] (Figure 1).

### 2.1. Synthesis of Pinosylvin in Plants

The first isolation of pinosylvin (3,5-dihydroxy-trans-stilbene) was performed in 1939, by V. H. Erdtman [47], in extracts of *Pinus silvestris*, hence its name; since then it has been identified in a number of plant species (Table 1). The common precursor molecule of all stilbenoids is phenylalanine or tyrosine, which are obtained by glucose metabolism, through the shikimate or arogenate pathways [48]. Pinosylvin, resveratrol, and other stilbenoids are classified as phytoalexins, and are synthesised by some plants in response to environmental stressors [49,50]. Both flavonoid and stilbenoid phytoalexins are derived through the universal phenylpropanoic–polymalonic acid pathway; endogenous and exogenous stimuli regulate the expression of these pathways [50]. Pinosylvin synthase, the key enzyme in the biosynthetic sequence, was isolated in 1984, using column chromatography [51]. A detailed description of stilbene biosynthesis is provided by Mendonça et al. [52].

**Figure 2:**
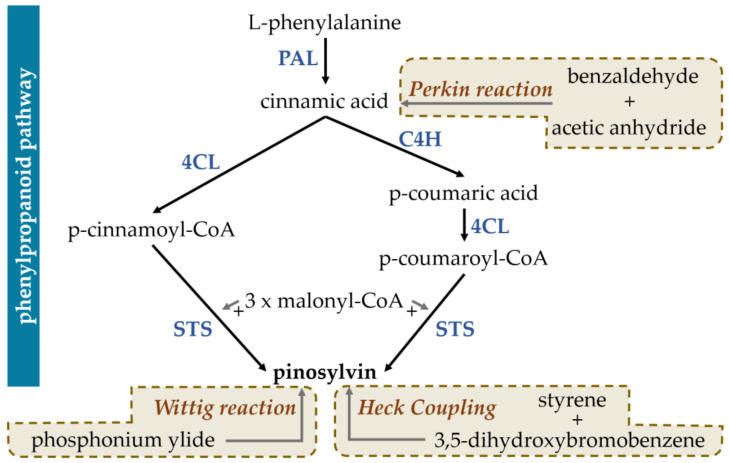
Schematic representation of biosynthetic (phenylpropanoid) pathways and chemical pathways (beige overlay) for pinosylvin. PAL = phenylalanine ammonia-lyase, 4CL = 4-cumaroyl: CoA-lyase, C4H = cinnamate-4-hydroxylase, STS = stilbene synthase.

A schematic of the biosynthetic and chemical pathways of pinosylvin synthesis is available in Figure 2.

### 2.2. Pharmacokinetics of Pinosylvin

Details on the metabolism of stilbenes and pinosylvin in particular are relatively unknown [82]; the particularities of the metabolism of different stilbenes hinder the development of a generalised understanding of their metabolism as a class [82,83].

A part of them is metabolised in the liver, via glucuronidation and sulfation; the other part is metabolised in the intestine [82,83,84,85,86,87]. Stilbene sulfation seems to be associated with their cardioprotective and anticancer activities [88]. Regarding intestinal metabolism, perhaps some elements can be inferred from related experiments on resveratrol, whose intestinal biotransformation peaks at about one hour post-consumption; however, hepatic biotransformation is much more important [89,90]. For resveratrol, the oral bioavailability has been found to be about 30% in rats, but in humans it is about 0.5% [91].

### 2.3. Methods of In Vitro Pinosylvin Synthesis

Pinosylvin does not naturally occur in appreciable amounts and so its isolation and purification by conventional methods requires a complex multi-step process, which is technically challenging and economically unsustainable [41]. Direct chemical synthesis also has similar drawbacks, coupled with the formation of dangerous by-products [92]. In the last few years, artificial biosynthesis of hydroxystilbenes and associated compounds has gained traction [93,94,95]. The currently available methods for pinosylvin synthesis are presented in Table 2 in chronological order.

The first method for artificial pinosylvin procurement was proposed by Jorgensen [96,97]; the method demonstrated that by causing mechanical damage to the stem and cambium of red pine, fungal penetration ensues and this leads to the synthesis of pinosylvin and its monomethyl ether. This method made possible a more detailed study of the biosynthetic pathway of pinosylvin, in plant cells in vitro [98]. So, in this case, pinosylvin is produced by the plant cells in response to desiccation. Improvements to this original scheme were soon proposed by Jorgensen and Balsillie [96].

Another approach is the production of pinosylvin from in vitro cell cultures of *Pinus strobus* L. cells in a specially modified culture medium [99]. It is also possible to induce pinosylvin accumulation in methanolic extracts, from *Pinus sylvestris* L. cell cultures by using an elicitor preparation of the spine needle pathogen [100].

Apart from these methods, which are centred on using plant cells, in one way or another, the production of pinosylvin using bacterial cells has been tried successfully [101]. Pinosylvin, as well as other stilbenes, has been produced by the genetic modification of *E. coli* as well by similar modifications on *Pseudomonas taiwanensis* and *Corynebacterium glutamicium* [102,103,104,105,106,107,108,109,110].

**Table 2:** Methods of artificial synthesis of pinosylvin and other associated stilbenes, by chrono-logical order.

Pinosylvin Producer	Method	Result	Year	References
Cells of *P. resinosa*	Stimulation of plant cells in response to desiccation	Production of pinosylvin and pinosylvin monomethyl ether	1961	[97]
Cells of *P. resinosa*	Stimulation of plant cells in response to desiccation	Production of pinosylvin and pinosylvin monomethyl ether	1969	[96]
*Pinus sylvestris* L. cells	Treatment of cells with an elicitor preparation from the pine needle pathogen *L. seditiosum*	Production of pinosylvin and pinosylvin-3-O-methyl ether	1994	[100]
Metabolically engineered *E. coli*	Construction of a pathway for stilbene biosynthesis inside *E. coli* cells	Production of stilbene polyketides	2006	[102]
Metabolically engineered *E. coli*	Construction and subsequent modification of a pathway for stilbene biosynthesis inside *E. coli* cells	Production of stilbene methyl ethers	2007	[103]
Metabolically engineered *E. coli*	Construction of a pathway for stilbene biosynthesis inside *E. coli* cells	Production of pinosylvin	2015	[104]
Metabolically engineered *E. coli*	Establishment of a variety of biosynthetic pathways in *E. coli* cells using enzymes from different sources	Production of various phenylpropanoid derivatives	2015	[105]
Metabolically engineered *E. coli*	Development of three different bioengineering strategies	Production of pinosylvin	2016	[106]
Metabolically engineered *C. glutamicium*	Construction of a pathway for stilbene biosynthesis inside *C. glutamicium* cells	Production of pinosylvin and other associated compounds	2016	[110]
Metabolically engineered *E. coli*	Construction and subsequent modification of a pathway for stilbene biosynthesis inside *E. coli* cells	Production of pinosylvin	2018	[107]
Metabolically engineered *E. coli*	Reduction of specific gene expression in order to increase pinosylvin production in already-modified *E. coli*	Production of pinosylvin (increased compared to the originally modified strain)	2018	[108]
Callus cells of *P. strobus* L.	Aging of callus cells in a specially modified culture medium	Production of pinosylvin stilbenes	2022	[99]
Metabolically engineered *P. taiwanensis*	Construction of a pathway for polyketide biosynthesis inside *P. taiwanensis* cells	Production of polyketides	2023	[109]

## 3. Pinosylvin as an Antibacterial Agent

In the last decades, plant metabolites have been steadily gaining ground as viable antibacterial agents which may help mitigate the problem of microbial resistance to antibiotics, that is caused both by the versatility and adaptability of bacteria—indeed, they survive in a host of adverse environments such as hot springs—and the overuse of antibiotics [29,111,112,113,114,115,116,117,118,119,120,121,122,123,124,125,126,127,128,129,130]. Apart from potential resistance, most common antibiotics may be associated with allergies, hepatoxicity, changes to normal microbiota, and other side-effects [131,132,133,134,135,136,137,138].

The spectrum of antibacterial properties of pinosylvin and some of its derivatives is quite broad, ranging from relatively unknown pathogens to potentially dangerous and resistant strains (Table 3).

### 3.1. Antibacterial Properties Against Achromobacter xylosoxidans

This pathogen is associated with nosocomial infections and notable resistance to antibiotics [148,149]. Medical water, particularly sterile distilled water bottles, is one of its main reservoirs [148]. It can cause a wide range of pathologies, such as ecthyma gangrenosum, bacteraemia, and pneumonia [149,150].

According to Lindberg et al. [60], the strain A50182 was affected by the presence of pinosylvin though not as much as the Gram-positive bacteria included in the study. At any rate, the extracts *P. sylvestris*, *P. resinosa*, and *P. contorta* were the most impactful, possibly due to the presence of stilbenes.

### 3.2. Antibacterial Properties Against Arcobacter butzleri

This bacterium is closely related to Campylobacter spp., shows increased resistance, and was recognized as an emerging pathogen in 2002 [139,151,152,153]. It can be found in many different types of food such as meat, dairy products, vegetables, and seafood and usually affects the digestive tract [151,153]. The increased resistance means that research should be directed at finding additional treatment options.

Pinosylvin was found to be effective against two strains of *A. butzleri*, namely DQ46M1 and CR50-2, at a concentration of 128 μg/mL [139]. Another beneficial aspect is the synergistic action it displayed alongside several antibiotics, as was made evident by the reduction in MIC [139]. Interestingly, the results of Sousa et al. [139] also suggest that pinosylvin could potentially even revert antibiotic resistance. The efficacy of pinosylvin seems to be related to its ability to interact with the activity of efflux pumps, inhibiting them [139].

### 3.3. Antibacterial Properties Against Bacillus spp.

#### 3.3.1. *Bacillus cereus*

This is a species of facultative anaerobic bacteria, which are most notable for their ability to form spores, thus becoming more resistant to a variety of factors [154,155]. *B. cereus* can induce significant mortality, especially when it affects the CNS [156,157]. It is commonly associated with ocular, respiratory, and wound infections, although it is most frequently encountered in contaminated food, such as in the case of the ‘‘fried rice syndrome’’ [157,158].

Pinosylvin was found to be potent in its action against *B. cereus* though the mechanism behind its significant effectiveness has not been elucidated [41,146]. In fact, Välimaa et al. [140] found it to be the most susceptible to pinosylvin alongside *L. monocytogenes*.

#### 3.3.2. *Bacillus coagulans*

This probiotic bacterium has been very useful in its application for medicinal purposes, most notably its beneficial role in several intestinal pathologies, like constipation, diarrhoea, colitis, and, most notably, irritable bowel syndrome [159,160].

There is evidence that showcases the effectiveness of pinosylvin against this bacterium, more specifically the strain E50L1, especially when compared with its effectiveness against Gram-negative bacteria [60]. This is likely a result of the stilbenes contained in the extracts; the extracts of *P. sylvestris*, *P. resinosa*, and *P. contorta* were the most potent in that regard.

#### 3.3.3. *Bacillus subtilis*

This bacterium not only is non-pathogenic but has also been studied extensively due to a multitude of factors, contributing greatly to our understanding of several biological aspects [161]. It has even been used as a probiotic, in order to regulate intestinal flora, and recently it is being looked into as a potential vaccine expression vector [162].

Pinosylvin is active against *B. subtilis* at a concentration of 64 g/mL [141]. This activity was corroborated by Bouarab-Chibane et al. [142]. It is presumed that the susceptibility of *B. subtilis*, as well as that of other Gram-positive bacteria, is somehow related to their membrane structure [163].

### 3.4. Antibacterial Properties Against Burkholderia multivorans

*Burkholderia multivorans* belongs to the *Burkholderia cepacian* complex, which is notable for its resistance to antibiotics [164,165]. This bacterium usually infects the lungs of patients suffering from cystic fibrosis, though it is also becoming an important cause of neonatal sepsis [164,165,166]; rarely, it may also cause bacterial meningitis [167].

The research of Lindberg et al. [60] found this bacterium to be susceptible to the action of pinosylvin, most probably because of the extracts’ stilbenes, though less so than the Gram-positive bacteria on which it was tested. From the extracts included in this research, *P. sylvestris*, *P. resinosa*, and *P. contorta* were the most effective. The specific strains which were found to be susceptible were F45L5 and F453DL1 [60].

### 3.5. Antibacterial Properties Against Campylobacter spp.

Both *C. coli* and *C. jejuni* can be isolated from poultry and poultry-related products [168]. Campylobacter spp., and *C. jejuni* in particular, are the leading cause of food-borne pathologies all over the globe [168,169,170]. The commonly associated condition is a usually self-limiting gastroenteritis [171]. However, it can also have serious consequences like miscarriage or even Guillain–Barré syndrome due to molecular mimicry [172,173]. *Campylobacter coli* can also cause severe conditions, albeit rarely, like bacteraemia, and recently, the first recorded case of infantile myocarditis [174,175]. Their resistance to antibiotics is an ever-growing concern that needs to be addressed [170].

Pinosylvin seems to be a promising agent against Campylobacter spp. [41]; the compound described by Silva et al. [144] exhibited colony count inhibition higher than 99% at a concentration as low as 0.08 mg/cm^2^. This research also demonstrated the effectiveness of coated pad-pinosylvin ICs in preventing Campylobacter contamination of fresh chicken. The effectiveness of pinosylvin in the case of Campylobacter spp. is attributed to its ability to damage the bacterial membrane, therefore acting in a bactericidal way [143].

### 3.6. Antibacterial Properties Against Escherichia coli

This bacterium makes up an important part of the physiological gut microbiota, but it may cause opportunistic infections, especially in the case of immunocompromised patients [176,177]. This becomes a significant problem in the wake of the appearance of multi-drug resistant (MDR) *E. coli* strains [178].

Pinosylvin was found to be effective in this regard, namely at a concentration of 250 µg/mL [145]; promising results were also discovered by Bouarab-Chibane et al. [142]. This effectiveness was corroborated by the findings of Hou et al. [141], though at a different concentration, namely 68 g/mL. However, related to the susceptibility of other bacteria, Välimaa et al. [140] concluded that pinosylvin is on the lower side of efficacy against *E. coli*.

### 3.7. Antibacterial Properties Against Listeria monocytogenes

These ubiquitous, intracellular bacteria are transmitted via the faecal–oral route and are the cause of foodborne pathologies, owing in large part to their ability to survive in refrigerator temperatures [179,180,181,182]. Infection with this pathogen can lead to severe complications like meningoencephalitis in immunocompromised patients and pregnant women and their foetuses, while healthy adults may experience the disease as a self-limiting gastroenteritis [179,180,181,182].

Pinosylvin derivatives have been found to be effective against *L. monocytogenes* [41,142,183]. It is at the storage temperature of 8 °C that pinosylvin exerts the highest effectiveness against inoculated *L. monocytogenes*, completely inhibiting its multiplication at a concentration of 140 μg/g [146]. As the temperature rose to 20 °C, the antibacterial effect was reported to last for 72 h, with bacterial growth becoming logarithmic and profuse after this temporal threshold [146]. These findings are corroborated by the research of Välimaa et al. [140] who found *L. monocytogenes* to be the most susceptible to pinosylvin alongside *B. cereus*.

### 3.8. Antibacterial Activity Against Proteus vulgaris

*P. vulgaris*, alongside *P. mirabilis*, are the most notable species of their genus. The disease most commonly associated with this bacterium are urinary tract infections, such as cystitis and pyelonephritis [184,185]. Alarmingly, *P. vulgaris* has been associated with resistant nosocomial infections [186]. Pinosylvin is active against *P. vulgaris* at a concentration of > 128 g/mL [141].

### 3.9. Antibacterial Activity Against Pseudomonas spp.

#### 3.9.1. *Pseudomonas aeruginosa*

*P. aeruginosa* is commonly implicated in nosocomial infections [187,188,189]. These infections range from urinary tract infections to pneumonia and can have significant morbidity and mortality, especially when the patient also suffers from COPD or cystic fibrosis [187,188,189,190,191,192]. An exacerbating factor is the polyresistance commonly encountered in the strains of this bacterium [187,188,189]. Pinosylvin is active against *P. aeruginosa* at a concentration of >128 g/mL [141]. This activity is corroborated by the research of Bouarab-Chibane et al. [142].

#### 3.9.2. *Pseudomonas fluorescens*

These ubiquitous bacteria can be found as part of the physiological microbiota on many parts of the human body and can occasionally cause opportunistic infections [193,194]. These infections range from bacteraemia to pneumonia or can even be ocular [18,194,195]. It is worth mentioning that drug-resistant strains are emerging [193], which is a cause for alarm. Pinosylvin has exhibited some potential as an agent against the growth of this bacterium, but not as notable as in the case of other bacteria [41,140].

### 3.10. Antibacterial Activity Against Salmonella spp.

*Salmonella infantis*, a serovar of *S. enterica*, has a zoonotic transmission and it is implicated in a significant number of foodborne infections worldwide, being mainly found in broilers and their meat [196,197]. Alarmingly, it presents with resistance to a wide range of antibiotics, including colistin which is among the last lines of defence, while also being highly virulent, this being attributed in large part to its biofilm-forming capacity [196,197]. These multidrug-resistant (MDR) strains have the potential to cause major health problems, especially in low- and middle-income countries [197].

Pinosylvin has exhibited some potential as an agent against the growth of this bacterium [41], though not nearly as prominently as in the case of other bacteria [140]. The research of Bouarab-Chibane et al. also highlighted the effectiveness of pinosylvin against *S. enteritidis*, another common serovar of salmonella associated with foodborne disease outbreaks [142,198].

### 3.11. Antibacterial Activity Against Staphylococcus spp.

#### 3.11.1. *Staphylococcus aureus*

This bacterium often colonizes the human body, though it is also the causative agent of several infectious conditions such as food poisoning, scalded skin syndrome, pneumonia, and bacterial endocarditis [199,200,201,202]. Staphylococcus is particularly notable in the clinical setting due to its implications in implant infections during orthopaedic surgery, as well as to the existence of particularly resistant strains, namely the methicillin-resistant *Staphylococcus aureus* strains, which have a very negative impact on patient morbidity and mortality [203,204,205,206]. Due to the emergence of strains with reduced susceptibility to vancomycin, one of the last lines of defence against this pathogen [206], extensive research in order to formulate effective treatment schemes is necessary [199].

Pinosylvin has been found to be effective in inhibiting *S. aureus* at concentrations of 75 μg/mL to 250 µg/mL, while prenylated pinosylvin was able to inhibit the growth of even MRSA strains at a concentration of 12.5 µg/mL [145,146,147]. The findings of Bouarab-Chibane et al. were similar, as were those of Hou et al., the latter settling on a concentration of 64 g/mL, and regarding MRSA, pinosylvin acid derivatives exhibited effectiveness at a concentration of 16 g/mL [141,142]. In any case, the data provided by Välimaa et al. [140] suggest that the action of pinosylvin against *S. aureus* is considered moderate when compared to its effects on other bacteria.

#### 3.11.2. *Staphylococcus epidermidis*

This bacterium can be found as a commensal on the human skin and occasionally in the nasopharynx [207]. Even though it has a beneficial role in maintaining the integrity of the skin as a barrier against infection, it can occasionally become pathogenic, especially when it comes to contaminating implants, and in a select few cases, it can even cause toxic shock syndrome [208,209,210,211]. Pinosylvin is active against *S. epidermidis* at a concentration of 128 g/mL [141].

## 4. Pinosylvin as an Antifungal Agent

In general, there are vastly fewer fungi which are pathogenic to humans compared to bacteria [212]. Even then, most fungal infections are not considered life-threatening, unless there exist particular risk factors. Be that as it may, fungal infections still represent an appreciable threat to human health, especially in some particular regions [213,214,215]. Just like in the case of bacteria, resistance is a potentiality [216,217,218], while some side-effects of antifungal drugs have also been noted.

The spectrum of antifungal properties of pinosylvin and some of its derivatives has not been tested as extensively as its antibacterial potential; nevertheless, the results are encouraging and justify further research on the subject (Table 4).

### 4.1. Antifungal Activity Against Aspergillus fumigatus

This is a ubiquitous saprophytic fungus [219]. Aspergillosis can be dangerous, particularly when it encounters a depressed immune system [219]. A notable case is that of allergic bronchopulmonary aspergillosis which affects patients with asthma or cystic fibrosis and can lead to bronchiectasis [220]. The emergence of triazole-resistant *Aspergillus fumigatus* strains is therefore a cause for concern [221].

There are studies that highlight the potency of pinosylvin against this microbe by Bakrim et al. [41] and Välimaa et al. [140]. The extracts tested in the latter study [140] were able to inhibit the germination and growth of the fungus.

### 4.2. Antifungal Activity Against Candida albicans

Candida spp. are often harmless but on occasion, particularly in the case of *Candida albicans*, they are capable of causing pathologies, most notably related to the vagina and the oral cavity, while also possibly playing a negative role in the exacerbation of chronic inflammatory bowel diseases [222,223,224,225]. Infections caused by this microorganism may be severe, leading to significant nosocomial complications [224,226,227,228].

Pinosylvin has potent antifungal effects against Candida albicans according to Barkim et al., Välimaa et al., and Lee et al., in the latter case, at a concentration of 62.5 μg/mL [41,140,145]. As noted in the work of Välimaa et al. [140], all Pinus extracts were effective in this regard, but the most prominent action was that exhibited by the extracts of *P. resinosa*, *P. strobus*, and *P. sibirica*. Pinocembrin, another flavonoid, is similarly effective though the mechanism behind this has not yet been elucidated [73].

### 4.3. Antifungal Activity Against Cladosporium herbarum

This microorganism has been known to occasionally cause hypersensitivity pneumonitis, a delayed allergic reaction [229,230]. Thankfully, barring the case of patients with compromised immunity status, they do not seem to be otherwise pathogenic [231].

Kostecki et al. [78] found that compounds with an unsubstituted aromatic ring A, like pinosylvin, exhibit notable antifungal activity, its EC_50_ being 10mg/mL. The findings of Pacher et al. [79] are similar and extend to four microfungi, namely *Alternaria citri*, *Fusarium avenaceum*, *Pyricularia grisea*, and *Botrytis cinerea*. Among them, *P. grisea*, a necrotrophic plant pathogen with widespread distribution that causes grey mould disease, was the most susceptible to the action of the stilbenoids [79,232].

### 4.4. Antifungal Activity Against Plasmopara viticola

This obligate biotrophic oomycete is the causative agent of the grapevine downy mildew [233], a major threat for grape cultures and wine production worldwide [233,234,235]. Its effectiveness as a pathogen is attributed to a great variety of pathogenicity effectors, and it has long since developed resistance to several fungicides [233,234,235].

The effectiveness of pinosylvin against this oomycete has been highlighted by Bakrim et al. and Gabaston et al. [41,73]. In particular, Gabaston et al. [73] assumed that the *Pinus pinaster* knot extract owes this effectiveness to its high concentration of pinosylvin, pinosylvin monomethyl ether, and pinocembrin. This claim is supported by the results of the bioassays of these molecules in the presence of *Plasmopara viticola* [73]. Based on this information, Gabaston et al. [73] suggest that the by-products of *Pinus pinaster* knot could be used in the field of viticulture as protective agents.

### 4.5. Antifungal Activity Against Penicillium brevirocompactum

This species of penicillium, alongside several others such as *P. expansum* and *P. digitatum*, is responsible for fruit spoilage, particularly when it comes to pears [236]. This has considerable implications as far as market profits are concerned [236]. As far as the field of medicine is concerned, in vitro models have exhibited the capacity of its metabolites to bring about inflammatory reactions and cytotoxic effects in mice [237]. In a similar vein, it has been proven to be a potent sensitizer for asthmatic patients [238].

Based on the writings of Bakrim et al. and Välimaa et al. [41,140], pinosylvin is a potent agent against *P. brevicompactum.* The extracts included in the tests of Välimaa et al. [140] were effective in inhibiting both the germination and the growth of this microorganism.

### 4.6. Antifungal Activity Against Saccharomyces cerevisiae

This microorganism has a host of applications, playing an important role in the production of fermentative foods and drinks, but also of biofuels [239,240]. Its genome has been domesticated and sequenced, allowing for many laboratory applications, such as the synthesis of an eukaryotic genome similar to its own [239,240]. From a purely medicinal point of view, it has been labelled as ‘‘generally regarded as safe’’ [240].

Pinosylvin has been found to be effective against this fungus according to both Bakrim et al., Välimaa et al., and Lee et al. [41,140,145]. In particular, Lee et al. [145] stated that the MIC is 125 μg/mL.

## 5. Pinosylvin as an Antiparasitic and Antiviral Agent

The data on the antiparasitic and antiviral properties of pinosylvin are scarce compared to the corresponding data on its antibacterial and antifungal properties.

Regarding its antiparasitic activity, pinosylvin monomethylether (PSM), alongside (−)-nortrachelogenin, were identified as nematicidal substances; the resistance of some pines, such as *P. strobus* and *P. palustris*, to the pinewood nematode *Bursaphelenchus xylophilus* is most likely attributed to them [71]. This is important because the parasite can cause pine wilt disease and has subsequently been quarantined in a number of countries following the application of strict protocols [241] due to extensive crop losses [242]. It should be noted that pinosylvin itself was also active against the parasite’s propagative larvae, though not to the same extent as PSM (LD_50_ of 4 ppm); this finding is one of several that suggest that a trans-type double bond conjugated with two aromatic rings and a hydroxyl group is necessary for nematicidal activity [71].

Regarding its antiviral activity, relevant research efforts were undertaken in the wake of the recent SARS-CoV-2 pandemic; this pandemic highlighted the highly contagious nature of the virus, and also had important psychological repercussions and effects on many people and age groups [243,244,245,246,247,248,249,250,251,252,253,254,255,256]. Though its main target is the lungs, where it brings about acute respiratory distress syndrome, the virus can find its way to several other parts of the body and has even been implicated in coagulopathies [250,257,258,259,260]. Due to the large size of its genome and its notable recombination capacity, it poses a challenge, as our understanding of the available effective therapeutic options is still developing [243,258,260].

The research of Naseem et al. [261] described the interaction of pinosylvin monomethyl ether as a ligand binding to the SARS-CoV-2 protein, Mpro. In this in silico model, the molecular docking study found out that this pinosylvin derivative was capable of binding to the protein, though not as effectively as other tested stilbene compounds [261]. Interestingly, an in vitro model testing synthesized stilbene derivatives found them to be similarly capable of inhibiting SARS coronavirus replication [262].

## 6. Antioxidant Properties of Pinosylvin

Pinosylvin, as well as its derivatives, possess a remarkable number of antioxidant properties exerted by different mechanisms (Table 5). There is evidence to suggest that the antioxidant properties of pinosylvin are associated with its anticancer and anti-inflammatory properties [263,264,265,266,267,268,269,270].

A number of researchers have identified the ability of such compounds to scavenge free radicals either in pure form or when present in extracts evaluated by specific in vitro assays [63,69,271,272].

The research team of Park et al. [266] investigated the effect of pinosylvin on the production of nitric oxide (NO), using lipopolysaccharide (LPS)-stimulated murine macrophage RAW 264.7 cells. It is known that NO is physiologically produced via a reaction involving nitric oxide synthase (NOS), which exists in three isoforms [273,274]. A deficiency of some NOS isoforms leads to disease [275,276], as well as excessive NOS expression [277]. An isoform of NOS, termed by the researchers as inducible NOS (iNOS), is stimulated, among others, by LPS, hence the treatment of the RAW 264.7 cells. It was found that the antioxidant effect of pinosylvin in this case was associated most probably with TRIF-mediated signalling, inhibition of iNOS directly, and via mRNA expression inhibition [266]. Toll/IL-1R domain-containing adaptor-inducing IFN-b (TRIF) and its associated signalling pathway are necessary for interferon and other pro-inflammatory mediator production [278].

The research of Jančinová et al. [267], who incubated human neutrophils with pinosylvin in the presence of different pro-oxidant stimuli, determined that pinosylvin could suppress oxidant generation, probably via the inhibition of protein kinase C; a very similar mechanism has been demonstrated for resveratrol-induced protein kinase C inhibition [279]. This enzyme has long been thought of as instrumental in redox modification, with an important role in health and disease [280,281].

Jeong et al. [268] used bovine aortic endothelial cells, harvested from descending thoracic aortas, and using various concentrations of pinosylvin and different incubation times, found that it exerted an anti-apoptotic, proliferative, remodelling, wound healing-inducing, and anti-atherogenic effect. All these effects stem from the antioxidant effect of pinosylvin, which is mediated through the NO generation pathway [268].

Moreover, it was found that antioxidative protection against oxidative stress can be conferred, via inducing heme oxygenase 1 (HO-1) in human retinal ARPE-19 cells, conferring protection against oxidative stress [282]. In general, HO enzymes are involved in heme degradation, utilising NADPH and oxygen [283,284,285]. Concurrent effects on modulating growth factors associated with macular degeneration and other retinopathies or neuropathies may be beneficial for addressing a variety of diseases involving imbalances between ROS generation and the antioxidant defence system [286,287,288,289,290].

The more recent experiment of Wang et al., conducted on mice, aimed to explore the effects of pinosylvin on male infertility caused by oligoasthenospermia [291,292]. It was found that pinosylvin increased epidydimal sperm concentration and motility and induced an increase in testosterone levels, by decreasing oxidative stress via activation of the nuclear factor E2-related factor 2-antioxidant response element (Nrf2-ARE) pathway, an intrinsic defence mechanism against oxidative stress, also implicated in the progress of neuroinflammation and neurodegenerative diseases [291,293].

In 2010, Macickova et al. [265] induced adjuvant arthritis in rats, a condition known to cause an increase in pro-oxidant compounds in blood and tissues [294,295,296]. Indeed, they determined that hind paw volume and the chemiluminescence of the affected joint were reduced, indicating reduced inflammation and pro-oxidant compound levels [265]. The induction of adjuvant arthritis in rats was also employed by Jančinová et al. [267], and the oral administration of pinosylvin had reduced both neutrophil number and oxidant formation, thereby reducing inflammation. A potent effect against adjuvant arthritis was also noted by Bauerova et al. [270].

Also, in rats with adjuvant arthritis, the administration of pinosylvin, either as a monotherapy or in combination with carnosine resulted in a decrease in most inflammatory markers; this effect is believed to be associated with the antioxidant properties of these compounds [269].

**Table 5:** Experiments on the antioxidant properties of pinosylvin and its derivatives.

Compound	Source	Test Type	Mechanism	Concentration	Administration	Year	Reference
Pinosylvin	n/a (pure compound)	In vitro—pulse radiolysis experiments	Free radical scavenging in pH values between 2 and 12	0.1 mM aqueous solution	n/a	2002	[271]
Pinosylvin	n/a (laboratory synthesis)	In vivo—rat model	Inhibition of neutrophil infiltration	n/a	oral daily dose of 30 mg/kg b.w. for 28 d	2010	[265]
Pinosylvin	n/a (pure compound)	In vitro—LPS-stimulated RAW 264.7 cells	(probable) TRIF-mediated signalling, iNOS and mRNA expression inhibition	39.9 μΜ (IC_50_)	Pretreatment of cells with pinosylvin before LPS stimulation	2011	[266]
Pinosylvin	n/a (laboratory synthesis)	In vitro—human neutrophils	(probable) inhibition of protein kinase C	14.16 ± 1.46 μΜ/L (EC_50_)	Incubation of cells with pinosylvin	2012	[267]
		In vivo—rat model	Reduction in neutrophilia and oxidants production	n/a	Oral daily dose of 30 mg/kg for 21 d		
Pinosylvin	n/a (laboratory synthesis)	In vitro—bovine aortic endothelial cells	Mediation of NO production	Various (depending on different experimental protocols)	Incubation of cells with pinosylvin	2012	[268]
Pinosylvin	n/a (pure compound)	In vivo—rat model	Reduction in pro-oxidative processes	n/a	Oral daily dose of 30 mg/kg b.w. per os for 28 d	2012	[269]
Pinosylvin	n/a (pure compound)	In vitro—human retinal pigment epithelial (ARPE-19) cells	Promotion of HO-1 expression	Various (depending on different experimental protocols)	Incubation of cells with pinosylvin	2014	[282]
Pinosylvin	n/a (laboratory synthesis)	In vivo—rat model	Promotion of hepatic and pulmonary NF-κB activation, increase in lung lipo-oxygenase and promotion of plasma antioxidant status	n/a	Oral daily dose of 50 mg/kg b.w. twice a week for 28 d	2015	[270]
Pinosylvin monomethyl ether, pinosylvin, pinosylvin dimethyl ether	*P. merkusii*	In vitro—free radical scavenging experiments	Uptake of reactive oxygen species	11.4–25.8 mg/L (EC_50_ for extract)	n/a	2015	[69]
Pinosylvin	n/a (pure compound)	In vitro—ORAC-FL, ABTS and FRAP assays	Free radical scavenging	Various (depending on the assay)	n/a	2017	[272]
Pinosylvin	n/a (laboratory synthesis)	In vitro—mouse model	Activation of the Nrf2-ARE pathway	n/a	Intragastric daily administration of 100 mg/kg b.w for 2 w	2020	[291]
Pinosylvin, pinosylvin monomethyl ether	*P. caribaea*	In vitro—antioxidant assays using DPPH and ABTS methods	Free radical scavenging (electron donation/cation scavenging)	17.25 ± 0.78 μg/mL (IC_50_ for extract)	n/a	2023	[63]

## 7. Anti-Inflammatory and Anti-Allergic Properties of Pinosylvin

In general, natural stilbenoids, as well as their synthetic analogues, are considered effective anti-inflammatory agents [297,298]; both pinosylvin and its derivatives are potent inhibitors of inflammation, as proven by the extensive testing performed in vitro and in laboratory animals (Table 6).

Perhaps the most important mechanism regulating the inflammatory response in humans is the cyclo-oxygenase 2 (COX) pathway [299,300]. This is a rate-limiting step in the synthesis of prostaglandins (PGEs) and thromboxane A_2_ (TXA_2_) [301,302]. Inhibition of COX-2 pathway will inevitably lead to reduction in inflammation [303,304].

The earliest experiments with pinosylvin and its derivatives regarding their anti-inflammatory role were performed by Park et al. [297]; it was demonstrated that pinosylvin and some of its derivatives were effective in inhibiting COX-2-associated prostaglandin (PGE) production [297,305].

Pinosylvin, apart from its direct COX-2 inhibition, also inhibits COX-2 indirectly, via the transcriptional inhibition of NF-κB and its associated pathway [306]; it is known that NF-κB-associated signalling is associated with COX-2 production [307,308]. Still relating to the production of inflammatory mediators, the research of Adams et al. [81] demonstrated the potential of pinosylvin and other stilbenoids to inhibit leukotriene biosynthesis. In recent years, leukotriene metabolism has been the focus of research, concerning therapeutical anti-inflammatory interventions [309].

The anti-inflammatory properties of pinosylvin, connected to its antioxidant properties, were also demonstrated by Park et al. [266], in an in vitro study, as presented in the previous section. It will be recalled that similar results linking the antioxidant activity of pinosylvin with its anti-inflammatory one have already been demonstrated [267,294].

Another link between the antioxidant and anti-inflammatory properties of pinosylvin and its derivatives was revealed by Laavola et al. [76]; reduced NO production and iNOS expression, NF-κB transcription, along with interleukin 6 (IL-6) and monocyte chemoattractant protein-1 (MCP-1) production. IL-6 is important in inflammation and host immunity, but its dysregulation is connected to chronic inflammation and autoimmune pathologies [310]; MCP-1 functions by attracting monocytes, thereby contributing towards immunological surveillance at a tissue level [311]. In the in vivo arm of the same research, the administration of pinosylvin in rats with λ-carrageenan-induced inflammation led to a reduction in paw oedema [76]. Similar results regarding the effect of pinosylvin on IL-6 and MCP-1 were produced by Eräsalo et al. [312].

Likewise, the release of TNFα and IL-6 were inhibited by pinosylvin, due to its ability to inhibit the JAK/STAT pathway; a molecular docking study found that pinosylvin is able to bind to the active site of the JAK2 protein [264]. This pathway has been linked to inflammatory, auto-immune, and stress-related pathologies [313,314].

The in vivo and in vitro research of Moilanen et al. demonstrated a dose-dependent and rapid inhibition of TRPA1, which, among other functions, is involved in pain and odour perception [315,316,317,318]. Pinosylvin monomethylether was found to be an excellent peroxisome proliferator-activated receptor gamma (PPARγ) activator and it also reduced IL-6 activation [53]; PPARγ expression was also enhanced in murine macrophages, promoting a resolution of inflammation [319]; a similar result on inflammation resolution was noted by Kwon et al. [320]. On the other hand, in the experiment of Modi et al., pinosylvin was found to downregulate PPARγ and C/EBPa. The former has various roles in inflammation and even auto-immune diseases [321,322,323]; the role of the latter is still a subject of research [324,325,326].

Another in vivo study, performed by Aalto et al. [57] using Drosophila melanogaster, found that pinosylvin and pinosylvin monomethylether administration can inhibit induced immune responses in the intestine of the fruit fly by a transient receptor ankyrin 1 (TrpA1)-dependent antagonism. This is a Ca^2+^-permeable, non-selective cation channel, found in certain cell types [327]; it is currently under research as a promising target in the treatment of inflammatory diseases, pruritus, asthma, and even Alzheimer’s disease [328].

Regarding the anti-allergic properties of pinosylvin, only a few researchers have concentrated on this. Labib et al. [56] isolated (Z)-pinosylvin mono methyl ether, (Z)-pinosylvin-3-O-b-D-glucoside, along with other compounds, from the leaves of *Agonis flexuosa*, and found that it had an affinity for the human histamine receptor, and thus a histamine release inhibition, although at the moment there are more promising phytochemicals in relation to this aspect [56]. Pinosylvin, extracted from *H. dulcis* Thunb., inhibited the release of IL-4, TNF-α, and PGE2 and the expression of IL-4, TNF-α, COX-2, NFKB1, and NFKB2 in a dose-dependent manner in RBL-2H3 cells treated with IgE [54]. All of these hormones and mediators are important in the process of allergic responses [292,329,330].

**Table 6:** Experiments on the anti-inflammatory and anti-allergic properties of pinosylvin and its derivatives.

Compound	Plant	Tested on	Mechanism	Concentration	Administration	Year	Reference
Pinosylvin (and other derivatives)	n/a (laboratory synthesis)	In vitro—LPS-stimulated murine RAW 264.7 cells	Inhibition of COX-2-induced PGE production	10.6 μΜ (IC_50_)	Pretreatment of cells with pinosylvin before LPS stimulation	2004	[297]
Pinosylvin, dihydropinosylvin	n/a (laboratory synthesis) and *S. tuberosa* (dihydrop.)	In vitro—activated human neutrophils	Inhibition of leukotriene biosynthesis	~50 μΜ (IC_50_)	Incubation of cells with test compounds	2005	[81]
Pinosylvin	n/a (pure compound)	In vitro—human THP-1 monocytes	Inhibition of LPS-induced NF-κB activation	Various	Incubation of cells with pinosylvin	2006	[306]
Pinosylvin	n/a (pure compound)	In vitro—LPS-stimulated murine RAW 264.7 cells	(probable) TRIF-mediated signalling, iNOS and mRNA expression inhibition	39.9 μΜ (IC_50_)	Pretreatment of cells with pinosylvin before LPS stimulation	2011	[266]
Pinosylvin, monomethylpinosylvin	*P. sylvestris*	In vitro—murine J774 macrophages	Decreased iNOS expression and NO production, decreased NF-κB transcription	13–15 μΜ, 8–12 μΜ (ΕC_50_)	Addition in fresh culture medium post-cell growth (for 72 h)	2015	[76]
		In vivo—male C57BL/6 mice	Reduction of paw oedema	100 mg/kg	Administered via intraperitoneal injection once		
Pinosylvin	*H. dulcis* Thunb	In vitro—RBL-2H3 basophilic leukaemia cell line	Inhibition of released and/or expressions of inflammatory mediators	5–20 μg/mL	Treatment of cells with pinosylvin for 1 h	2015	[54]
Pinosylvin	n/a (pure compound)	In vitro—HEK293 (human embryonic kidney) cells	Inhibition of TRPA1 activation	0.1–100 μΜ (IC_50_ = 16.7–26.5 μΜ)	Pre-incubation of cells with pinosylvin	2016	[315]
		In vivo—male C57BL/6N mice	Reduction of IL-6 in inflamed tissue	10 mg/kg	Intraperitoneal injection (pinosylvin dissolved in 250 μL of phosphate buffered saline solution)		
Pinosylvin monomethylether	*C. cajan*	In vitro—LPS-stimulated murine RAW 264.7 cells	Activation of PPARγ and inhibition of IL-6 activation	Various IC_50_ values	Incubation of cells with solution containing the target compound	2016	[53]
Pinosylvin	n/a (pure compound)	In vitro—mouse 3T3-L1 preadipocyte fibroblasts	Downregulation of PPARγ and C/EBPa	116.8 ± 7.5 μΜ (ΕC_50_)	Incubation of cells with pinosylvin	2017	[331]
Pinosylvin, monomethylpinosylvin	n/a (laboratory synthesis)	In vitro—murine J774 macrophages	Inhibition of PI3K/Akt activation and of IL-6, NO, and MCP-1 expression	Various ^1^	Incubation of cells with pinosylvin	2018	[312]
		In vivo—male C57BL/6 mice	Reduction in carrageenan-induced paw oedema via inhibition of IL-6 and MCP-1	30 mg/kg	Intraperitoneal injection 1 h prior to inflammation induction		
Pinosylvin	n/a (pure compound)	In vitro—human THP-1 monocytes and human U937 cells	Promotion of leucocyte apoptosis via upregulation of ALOX15 expression	Various ^1^	Treatment of cells with pinosylvin	2018	[320]
(Z)-pinosylvin mono methyl ether, (Z)-pinosylvin-3-O-b-D-glucoside	*A. flexuosa*	In vitro—U937 human monocytes	Inhibition of histamine release	Various (less than the IC_50_ of ciprofloxacin) ^1^	Incubation of cells with target compounds	2020	[56]
Pinosylvin, monomethyl pinosylvin	n/a (pure compound)	In vitro—murine J774 macrophages	Downregulation of classical M1 macrophage activation and upregulation of alternative M2 activation	10, 30, 60 μΜ	Addition of target compounds in fresh culture medium after differentiation of monocytes to macrophages	2021	[319]
Pinosylvin, pinosylvin monomethylether	*P. abies*, *P. sylvestris*	In vivo—*Drosophila melanogaster*	1 (TrpA1)-dependent antagonism of NF-kB-mediated intestinal immune responses	100 μΜ or 500 μΜ	24 h feeding of larvae of indicated concentrations mixed with fly food	2023	[57]
Pinosylvin	*P. nigra laricio* var. *calabrica*	In vitro—LPS-stimulated murine RAW 264.7 cells	Inhibition of TNFα and IL-6 expression, via inhibition of the JAK/STAT pathway	40 μΜ (IC_50_ = 10.6 μΜ)	Pretreatment of cells with target compounds	2023	[264]

^1^ depending on different derivatives, experimental protocols, or tested in increasing concentrations for different actions.

## 8. Anti-Cancer Properties of Pinosylvin

Stilbenes, stilbenoids, and their derivatives have a host of anticancer properties [332,333,334,335,336,337,338]. Studies have demonstrated the antiproliferative properties of pinosylvin in particular, via inhibition of protein uptake, suppression of Src/ERK and GSK-3/β-catenin signalling, gene, metabolic and signalling modifications [339,340,341]. The Src/ERK pathway is associated with glucose metabolism and the metabolic plasticity of cancer cells [342], while GSK-3/β-catenin signalling is involved in a number of diverse pathways [343]; their suppression leads to stoppage of cells in the G_0_/G_1_ phase, as shown by Park et al. on human colorectal HCT 116 cancer cells [340].

Mellanen et al. determined that there was an increase in oestrogen expression and proliferation in certain breast cancer cells, when exposed to pinosylvin; in general, the expression of oestrogens and their receptors is an important component in breast cancers [344,345]. Several pinosylvin derivatives have shown effectiveness against MCF-7 breast cancer cells with pinostilbene being among the highest effective compounds [346].

A number of experiments have demonstrated the antimetastatic potential of pinosylvin and its derivatives, which mostly occurs via matrix metalloproteinase inhibition and/or downregulation of their expression, both in vitro and in vivo [263,347,348]. The downregulation of the ERK1/2 signalling pathway observed by Chen et al. on SAS and SCC-9 oral cancer cells is also noteworthy, as this is an important pathway in cancer as well as in other pathologies [263,349]; a downregulation of Akt phosphorylation was noted in the same research, with crucial consequences on tumorigenesis [350]. In addition, vimentin is highly expressed in metastatic cancers and correlates with poor patient prognosis, cadherins and zonula occludens (ZO) are important in cellular adhesion [351,352,353]; as demonstrated by Chuang et al. [348], pinosylvin decreases the expression of all these proteins. Finally, Du et al. determined that migration, in squamous cell carcinoma cells, is inhibited by regulating of the STX6/ITGA3/VASP pathway, which has a demonstrated association with tumour development and migration [354,355].

Pinosylvin is also characterised by a notable cytotoxic potential, which however applies both for healthy and cancer cells, albeit healthy cells have a higher tolerance [356]; the cytotoxic potential of pinosylvin is exerted by different mechanisms, as presented by Song et al. on THP-1 and U937 leukaemia cell lines, notably a downregulation of AMP-activated protein kinase α1 (AMPKα1), a central regulator of energy homeostasis [357,358]. Finally, effects of a chemopreventive nature, relating to COX-2 inhibition and antioxidant properties have been reported by Park et al. [359]. An overview of experiments on the anti-tumoral properties is provided in Table 7.

## 9. Neuroprotective Properties of Pinosylvin

The neuroprotective properties of stilbenes are mostly based on their anti-inflammatory and antioxidant properties [52]; relevant research already exists for resveratrol [360,361,362]. Current evidence is in favour of stilbenes in cases of neurodegenerative diseases [363]. Xu et al. specifically targeted the neuroprotective properties of pinosylvin [364] (Table 8).

In stroke and cerebral ischaemia, the subsequent reperfusion, if successful, must be accompanied by some form of neuroprotection, to avert neural cell death [365,366,367]. Pinosylvin was demonstrated as capable of reducing LDH levels, decreasing TUNEL-positive cells (i.e., where DNA fragmentation occurred [368]), and downregulating cleaved caspase 3 levels in PC 12 cells found to enhance Bcl-2 expression and decrease Bax expression. The alteration of mitochondrial function is associated with the pathological effects of cerebral ischaemia and contributes to neuron death [369,370]. Finally, pinosylvin activated the Nrf2 pathway, thus reducing oxidative stress and, thereby, mitochondrial dysfunction. In vitro, pinosylvin administration in rats improved the brain deficit and reduced the infarct volume [364].

## 10. Traditional Medical Applications of Pinosylvin-Producing Plants

A wide variety of plant species from the Dipterocarpaceae, Cyperaceae, Gnetaceae, Pinaceae, Leguminosae, Vitaceae families, and others produce stilbenes [371]. Such plants and their products have been included in the diets of numerous populations since ancient times and used as sources of drugs or as medicines themselves prominently at least until the 16th century in most cultures [372].

For pine trees in particular, pine nuts have been discovered in various cases in Nerja (Málaga) and Lattes in southern France, where the sites are dated to the Mesolithic period, indicating the importance of pine nuts in the diets of early humans [373]. The taste of pine nuts was mentioned by both the Ancient Greeks and Romans, and Roman legions carried pine nuts among their provisions [374]. We must note here that apart from plant parts and extracts, drinks and foods containing stilbenoids have long been used in folk medicine. For example, in Greece of the early 20th century, Malaka wine (from Malaga, Spain) was used as the basis of a medicinal elixir [375]; stilbenes, stilbenoids, and other phenols are detected in most wines [376,377]. Grapes have also been consumed since antiquity and are noted as having numerous health benefits [378]. Peanuts, another source of stilbenoids, and other bioactive compounds are considered an excellent functional food [147,379].

Stilbenes are included in many preparations of traditional medicine systems, such as Ayurveda and Japanese traditional medicine [380]; pinosylvin-producing plants have a prominent role in the pharmacopoeias and traditional medical systems of many cultures. In this section, we will provide a representative selection of the traditional uses of pinosylvin-producing plants, organised by region, and cross-reference the recorded uses against experimentally proven effects (Table 9).

### 10.1. Traditional Uses in Europe

In general, Greece, as a region, is characterised by an astonishing variety of plant diversity, and particularly of endemic plant species [413,414]. Accordingly, the most important doctors of Ancient Greek medical tradition, Hippocrates, Theophrastus, and Dioscorides, all focused on plants as the basis of their therapies [415,416,417,418,419,420].

Hippocrates refers to pine as an ingredient in an ointment meant to help with the healing of burns and possibly with lessening the post-burn scar [421]. The bark of pine trees was also mentioned by Dioscorides as being a treatment for burn wounds [421]—the antiseptic alkaloids found in pine bark are useful in that regard as confirmed by modern research [422,423]; pine nuts are also mentioned in the context of relieving chest infections [424]. Earlier references, from an Ancient Egyptian medical text, also mention pine nuts as a drug [425]. Galen, the most important physician of the Imperial Roman era [426]—his medical training was based on Ancient Greek teachings—also mentions the use of pine as an expectorant [424]. A similar action is described much later by Abu Ali al-Husayn ibn Sina, a Persian polymath of the 10th-11th centuries A.D., in his book *Canon of Medicine* [424]. Remarkably, it was also described by a rather more obscure Ancient Greek physician, Metrodora, in her book, *On Women’s Diseases*.

Moreover, parts of the pine tree had other uses, linked to religion and magic; for instance, Maenads, the female followers of the god Dionysus, would use a decoction containing pine and ivy fruits, along with other ingredients, to reach an altered mental state [427,428,429]. Such is the importance of the pine tree in Ancient Greek culture that pine cones have been found depicted in Ancient Greek coinage [429,430,431], and a pine wreath was awarded to the winner of the Isthmian games [432], an important ancient agonistic festival [433]. Pine nuts are also used in some local Greek culinary traditions and for wine storage [429].

In Italy, *Pinus halepensis* Mill. is found around the Mediterranean [434]; it is an evergreen tree which is found in arid and semi-arid climates at low altitudes [435]. It must be noted that the production of bioactive compounds of this plant, and hence its medicinal properties, depend upon the particular regional climate conditions [68]. The leaves of the plant are used in the treatment of various respiratory pathologies [436,437,438]; the resin of the buds has been used to aid in wound healing [439]. Based on the current evidence, the plant possesses a notable antioxidant activity [440,441,442,443,444,445,446,447,448], which may be associated with its wound-healing properties. The anti-inflammatory properties of the plant have also been investigated [446,449,450,451,452].

In another region of the country, namely Valfurva, the extract from *Pinus mugo* Turra is said to be used as an expectorant, although this property has not been verified using modern methods [405]. This specific Pinus species, which also grows in some other European countries like Romania, has been evaluated for its antifungal and antioxidant activities, which are promising according to the research of [453], although to the knowledge of the authors, no study has yet evaluated its pinosylvin content. Given the high pinosylvin content of other Pinus species, it seems reasonable that such content must be commensurate to that of the others.

Along with the Romans, the Dacians are part of the national identity of modern Romania [454]; Dacian medicinal knowledge was well-known and respected in the ancient world, and, indeed, many of the phytochemical traditions of these ancient people have carried over to traditional Romanian practices [455,456,457,458,459]. It seems that based on current knowledge, pinosylvin-producing plants were not prominent in their ethnopharmacological tradition.

However, in the regions of Romania formerly occupied by the Habsburg Empire, a number of pharmaceutical products derived from pine trees are mentioned [403]. Especially in Transylvania, plant-based pharmaceutical products are quite common in the medical tradition of the region [460,461,462]. Indeed, there is ample ethnobotanical research on the traditions and effectiveness of such remedies [403,463,464,465]. Both *Pinus nigra* and *Pinus sylvestris* are identified by a variety of terms by those inhabiting the regions [466,467,468,469]. *Pinus nigra* is used in the treatment of colds, cough and other respiratory complaints, furuncles, and warts [464,465,470,471,472,473,474]—the soot, needles or cones of the plant may be used depending on the occasion. The resin is also used to treat teeth decay, or, alternatively, for teeth cleaning [472,473,475]. Some reports also mention the use of parts of the plant for digestive complaints, [476], while a ritual use to ward off evil is mentioned in some Serbian regions [477].

The soot of *P. sylvestris* is used to treat asthma, coughs, and respiratory diseases [478], along with rheumatism [471,479]. The bark of the plant has also been mentioned as effective in the case of varicose veins [480]. Pine species were also used in ethnoveterinary practices [471].

Finally, in Spain, the inflorescence of *P. halepensis* Mill is used to treat asthma while leaf decoctions and infusions are used to treat colds; other parts of the plant are used to treat pain and baldness [481]. In other parts of Spain, various parts of the plant are used for a number of remedies including warts, the characteristic lesions caused by the human papilloma virus (HPV) [482,483]. Other uses of Aleppo pine’s parts and gum include expectorant and anti-abscess applications [484]. In the Balearic islands, the buds of the plant are used to treat bronchitis [485].

### 10.2. Traditional Uses in Africa

In Algeria, parts of *P. halepensis* Mill are used as a remedy for gastrointestinal system pathologies and as a disinfectant and antifungal agent [486,487]. Recent research has confirmed the antibacterial and antifungal [445,448,488,489,490,491,492,493,494,495,496,497,498,499] activities of the plant. In association with the treatment of the aforementioned gastrointestinal system pathologies, the cytoprotective properties of *P. halepensis* Mill extracts have been confirmed for hepatic and renal cells [500,501]. Compounds found in the plant have also been shown to be able to protect cellular DNA from damage and associated mutations [68,447].

A fruit infusion of the plant is used to treat haemorrhoids, ulcers, pulmonary pathologies, and tuberculosis [502]. In other regions of Algeria, parts of the plant are used for similar purposes, and also for renal inflammations [503,504,505]. A notable use is against delusional parasitosis, a mental condition, and as an adrenal gland stimulant [506,507,508].

In Morocco, the bark of *P. halepensis* Mill is widely used as a poultice to treat injuries, scars, and infections, and as an astringent [509,510,511,512]. The resin of the plant, along with its bark, is used in SW Morocco to treat pathologies of the digestive, integumentary, circulatory, and urogenital system [513]. Regarding circulatory system pathologies, extracts of the plant have a proven anti-coagulant and anti-haemolytic activity [442,446,447,514,515]. Although no anticancer use is specifically mentioned in medical traditions, it may be assumed that some of pathologies of the aforementioned systems may be associated with neoplasias; plant extracts have been found to possess a cytotoxic activity against human glioblastoma cell lines [516], human myeloma, and adenocarcinoma cells [517].

Notably, a bark decoction is used in NW Morocco to treat tuberculosis [518]. Infusions from the leaves of this plant are used in different parts of Morocco to treat infections and eczema [519]. A leaf decoction is also used against toothache [520,521]. Finally, in Nigeria, extracts of *A. hypogaea* are used as antidiabetic and anti-cholesterol medications, in cardiovascular pathologies, to promote weight loss, and even in cases of cancer [522,523] by the locals.

### 10.3. Traditional Uses in Asia

Traditional Chinese medicine (TCM) began developing in Ancient China, although at the moment, the exact timing of the formulation of its first theories is not entirely clear [524,525]. Regardless, even since the beginnings of these practices, medicinal plants had a prominent role [372,526].

*Arachis hypogaea* (peanut) is an important part of TCM; although initially believed to have been domesticated in Bolivia and Argentina and cultivated by the Incas, it was subsequently transported to Spain and Europe and then to Asia [527,528]. It is now considered to be amongst the most-cultivated crops worldwide. Peanut seed oil contains numerous phospholipids of medical significance and other bioactive compounds [529]. Stilbenes have been identified in different organs of the plant [381]. Stem and leaf extracts of the plant are used to treat sleep disorders, and it has been proven that such extracts can improve sleep behaviour in phenobarbital-treated rats [382,530,531,532]. The skin of the peanut is also used to treat haemorrhage and bronchitis [533]; the extract of the coat of the seed has been shown to have an antibacterial potential [383].

Parts of *H. dulcis* are used in China, as well as in Japan and Korea (discussed later on), as nutraceuticals and supplements [389]. A wealth of different research evidence, from animal experiments, suggests that the extracts of the plant have an anti-inflammatory, analgesic, and anti-allergic activity, use as a laxative, and a potential anti-osteoporotic effect [534,535,536,537,538,539,540,541,542]; it also seems to promote lipid metabolism [543,544,545]. The leaves of another plant, *Cajanus cajan*, are used as an analgesic to stem the flow of blood and to treat parasitic infections [387].

Korea has a rich medical tradition, which may be traced back to the medical systems of China and Japan [546]; for at least seven centuries, Korean traditional medicine (KTM) has existed as a distinct medical tradition, with its own therapeutical approaches and methods [547]. KTM is well-known for the use of herbs and plants as therapeutical agents, alone or in combinations and different modes of preparation [548,549,550].

Extracts of *Hovenia dulcis* have long been used for alcohol detoxification, and based on research evidence, it is indeed a property of extracts of the plant to lower blood alcohol concentrations [551,552,553,554,555,556,557]. It is believed that the extracts of the plant indirectly upregulate alcohol dehydrogenase and acetaldehyde dehydrogenase, the two enzymes involved in the alcohol metabolism pathway. On a related note, it has been proven that extracts of the plant have a hepatoprotective action, in chemically induced liver damage [555,558,559,560]. Given that free radicals are known to be a main cause of hepatic injury [561,562], the antioxidant capacity of the plant and of pinosylvin and its derivatives, along with other phytochemicals, in particular, appears to be in play here; in two experiments, glutathione-S-transferase activity was also upregulated [552,554]. Although some extracts of this plant have a demonstrated antidiabetic activity, this does not seem to be related to their pinosylvin content [388]. *Pinus densiflora* Sieb. et Zucc. has been widely used in the treatment of hypertension, atherosclerosis, stroke, diabetes, cancer, and balding [400].

In Pakistan, *P. roxburgii* Sargent is found in areas where the monsoons do not penetrate. Its resin is used as a stimulant, and it is also applied externally in cases of suppuration and swollen lymph nodes. Both the wood and the oleoresin of the plant are used to treat snake and scorpion bites; the oil of the plant is also used to cure gastrointestinal complaints and is considered a prokinetic [407,563].

On the Asiatic side of Turkey, the Turkish red pine (*P. brutia* Ten.) is found mostly in coastal areas, is mostly exploited for pine resin production [564], and, in many cases, for turpentine production [565,566]. In the region of the Kazdağ mountain, in Western Turkey, the resin of the plant, alone, or in combination with honey, is used to cure gastrointestinal complaints, as an anti-cough medication, and also to treat diabetes [395]. In Turkish folk medicine, other uses of the plant have been described, namely as a treatment of haemorrhoids and as a tonic [395]. A combination of resin and hot water is also applied externally for wound treatment [395].

The bark extract of *P. brutia* has been tested in combination with Pycnogenol^®^, and when applied on cotton fabrics, has a proven antibacterial activity against *Aspergillus brasiliensis*, a member of the black aspergilli section [567], and also promotes wound closure. This result indicates a promising research avenue in the development of eco-friendly natural antifungal finishes for medical textiles [397]. The antibacterial action of the extract in vitro, against *Staphylococcus aureus*, Bacillus cereus, *Listeria monocytogenes*, *Salmonella Typhimurium*, and *Escherichia coli*, was verified by the research of Erol et al. [399], who also noticed an interesting antiproliferative potential against certain cancer cell lines. Interestingly, the tar of the roots of the plant also possesses an antimicrobial potential [568].

Moreover, the cone extract of the plant has demonstrated a potential for preventing acute lung injury in certain concentrations [398]; the same combination has exhibited a potent anti-inflammatory potential in rat models [394]. The antioxidant potential of the extract of the plant has already been noted by Cretu et al. [396]. Finally, the extract of *P. brutia* along with that of other plants from the same family is of interest to the cosmetics industry [569].

In the Himalayas, *P. roxhburghii* Sargent, a native plant, contains pinosylvin and pinosylvin monomethyl ether in its bark, stem, and needle extracts [570,571]; it has various uses in the local medical systems, as well as in Ayurveda [75]. Extracts from various parts of the plant have demonstrated antibacterial activity against *Agrobacterium tumefaciens*, *E. coli*, *Salmonella arizonae*, *S. typhi*, *B. subtilis*, and other pathogens [572,573,574]; bark and needle extracts were also found to have notable antioxidant activity, analgesic activity, anticonvulsant activity, and anti-asthmatic activity [75,409,575,576,577]. Some marketed preparations of the plant are also available [578,579,580]. Wood oil from the plant also has a hepatoprotective activity, although pinosylvin has not been detected in it [581]. Finally, the plant also has some cultural uses [409]. Another widely used plant is *C. cajan*, especially for pathologies of the oral cavity, as a laxative [582], to treat intoxications, and also to induce lactation [583]. Other uses of the plant have been recorded in regions of Bangladesh and Trinidad and Tobacco [387].

### 10.4. Traditional Uses in North America

The regions of North America, in what is now Canada and USA, have had a long history of habitation before the arrival of European settlers [584]. In accordance with the norms observed in most other ancient civilisations, Native Americans relied mostly on plants and their extracts for treating the majority of ailments and pathologies [412].

In the British Columbia region of Canada, *Picea glauca* Voss is used for a variety of reasons. It is burned so that the smoke can clear the air of infectious agents; it is mixed with bear grease and applied on wounds as a healing ointment; in decoction form, it is used for sore mouths or it is mixed with fat and swallowed in cases of haemoptysis; finally, young buds of it are chewed in cases of strep throat and it is applied as a dab in the nostrils in cases of sinusitis [390]. The Carrier people are Native Americans, which have a long medicinal tradition, having identified and included in their medical practices a great variety of plants, of which they frequently use all parts [585,586,587,588].

Apart from the Carrier people, there are other indigenous people in the Canadian boreal forest, who use *P. glauca* extracts and ointments to treat chronic pain of various causes [393,589]. The pitch preparations of *P. glauca* have a proven antibacterial activity against *E. coli*, *S. aureus*, *P. aeruginosa*, *C. albicans*, and *A. fumigatus* [391]. Moreover, it seems that the extracts of different organs of the plant can be used to protect organs from glucose toxicity or deprivation [590].

Another tribe of Native Americans, the Cherokee, have an important ethnomedical and ethnobotanical tradition [591,592,593,594]. *P. virginiana* Miller grows from Pennsylvania to Alabama [595,596] and was widely used by the Cherokee Native Americans to produce a type of wash for sores and skin ulcers, with the sap of the plant being employed in wounds refusing to heal. The oil of the plant was used to bathe painful joints and a tea from the needles was used in cases of colds and fevers [412]. Based on the current evidence, although notable bioactive compounds are found in parts and extracts of the plant, there is no appreciable antimicrobial activity [412].

## 11. Discussion

All pine trees have been found to contain a very high amount of polyphenols and are, in general, high in minerals and nutritional value [597,598,599,600,601,602,603]. Of course, pinosylvin and its derivatives are present in other plant species, in lower quantities, as evidenced by the successful small-scale extractions of various researchers. Novel solutions for artificial pinosylvin synthesis are available [99,106,108,110] which may prove more feasible and cost-effective on a larger scale.

### 11.1. Health-Related Properties of Pinosylvin and Future Research Perspectives

Based on the current research evidence, the antibacterial, antifungal, antiparasitic, and antiviral properties of pinosylvin and its derivatives are notable in in vitro settings [41,71,140,261]. Given the potential for complications of certain bacterial infections like tuberculosis [604,605], antimicrobial applications represent an important aspect of phytochemical research. This is important both in the context of emerging bacterial and fungal resistance to antibiotics, the prevalence of parasitic infections, and the increasing risk of viral pandemics [606,607,608,609,610,611,612]; the septic potential and the significance of sepsis in clinical settings must also not be ignored [613,614,615,616]. Moreover, considering the possibility of adverse effects, in antibacterial, antifungal, and antiviral, and especially antiparasitic drugs, pinosylvin formulations should be developed as adjuvant antimicrobial treatments to lower the dose or length of drug administration, if and when needed [134,617,618,619,620,621,622,623,624,625].

The antioxidant properties of pinosylvin in scavenging free radicals [69,267,271] are well demonstrated. Apart from that, the antioxidant effects can be exerted via other mechanisms and pathways [268,282]. The antioxidant properties of pinosylvin and its derivatives are also related to its anti-inflammatory and anticancer properties [263,269,270]. Considering the importance of oxidative stress in health and longevity, and its relevance in the pathophysiology of different diseases [626,627,628,629,630,631], relevant uses of pinosylvin should be explored; additionally, the use of pinosylvin as a supportive treatment in heavy metal poisoning, given that one of the mechanisms of metal toxicity is oxidative stress induction, could demonstrate its utility [632,633].

Regarding its anti-inflammatory potential, pinosylvin is both a direct and an indirect inhibitor of COX-2 [297,306]; it also inhibits leukotriene biosynthesis directly [81]. Other anti-inflammatory mechanisms of pinosylvin include IL-6 and MCP-1 inhibition [312], inhibition of the JAK/STAT pathway [264], and PPARγ expression upregulation [319] (TrpA1)-dependent antagonism [57]. A couple of research studies [56,535] have commented specifically on the anti-allergic potential of pinosylvin and its derivatives and have demonstrated an anti-inflammatory effect in the intestine of *D. melanogaster*. While such an effect has yet to be translated into applications in humans, it could be a promising therapeutical application for patients with chronic intestinal inflammation, such as inflammatory bowel syndrome patients [634,635,636].

Regarding the anticancer properties of pinosylvin and its derivatives, there is a noted and quite important antimetastatic potential [347]. The related anticancer effects of pinosylvin are mediated by a variety of mechanisms [348,354]. The cytotoxicity of pinosylvin is another potential anticancer action although the toxic effect is relatively important for healthy cells too [356]. Chemopreventive effects were also noted by Park et al. [359]. Direct antiproliferative properties are exerted by a number of mechanisms [339,341]. With the exception of the in vivo arm of the experiment of Park et al. [347], all other experiments mentioned in the text were performed in vitro. Given that some traditional medical uses of pinosylvin are mentioned for ocular pathologies, perhaps pinosylvin may offer a viable therapeutical option in cases of ocular cancers, such as uveal melanoma [637,638,639]. Therefore, while considering the promising anticancer potential of pinosylvin and its derivatives, the distribution, bioavailability, and effective concentrations at target cites remain to be solved. Considering the multifactorial nature of cancer pathogenesis and development [640,641,642,643,644,645,646,647,648,649,650], the interplay between pinosylvin and different molecular mechanisms should be further explored.

On the subject of distribution and bioavailability, we should mention that current research efforts on novel delivery systems may go a long way towards simplifying administration-related complications. In recent years, liposomes, micelles, micro-emulsions and nano-emulsions, colloidal capsules, and solid nanoparticles have been developed, and these may be used as carriers for pinosylvin and its derivatives [651,652,653,654]. The introduction of pinosylvin in 3D-printed biomaterials is another option, considering the rapid developments in the field of orthopaedic surgery, and the associated risk of infections [203,655,656,657,658,659,660,661,662,663,664,665,666]. Such research is all the more important considering also the advances in imaging techniques [667,668,669,670,671,672,673] which allow for earlier diagnoses and more complex surgeries. As of yet, there do not exist detailed studies examining the toxicology of pinosylvin, in a manner similar to other phytochemicals, such as capsaicin [674]. Few researchers have examined the toxic doses of pinosylvin at a cellular level, with the toxicity limit being above the effective limit; the relevant research results are summarised by Bakrim et al. [41].

Apart from the heretofore presented properties of pinosylvin, other health-promoting effects have been noted. For example, it was found that pinosylvin can inhibit adipogenesis in murine adipocytes [321]; considering the deleterious health effects of obesity (e.g., [675,676,677,678]), further research on the anti-adipogenic effects of pinosylvin should be undertaken. A number of studies point towards the antidiabetic effects of stilbenes [52]; pinosylvin has been proven capable of reducing insulin resistance in rat skeletal muscles [331], and this is a promising research avenue for future in vitro research in human cells. It is not clear yet if pinosylvin can have a meaningful antidiabetic effect, but considering the diabetes-associated complications [679,680,681,682], and the existing evidence on the antidiabetic effect of certain stilbenes [683,684,685], future research in that direction seems to hold some potential.

Currently, no specific cardioprotective effects of pinosylvin have been identified. This is in contrast to other stilbenes and their analogues [686,687,688]. For example, resveratrol is known to exert cardioprotective effects by modulating cholesterol levels, impacting cardiovascular risk [689,690,691,692,693]; pinosylvin, which has similar properties, is very likely to have commensurate effects. Additionally, it has been demonstrated that it prevents necrosis in bovine aortic endothelial cells at sufficiently high concentrations [694].

As mentioned, parts of *P. roxhburghii* Sargent have hepatoprotective properties [581]; this might be partly attributed to the presence of pinosylvin. Already a number of phytochemical compounds with hepatoprotective action, including stilbenes, have been identified [695,696]. Pinosylvin, its analogues, and derivatives, if proven useful in that regard, could be administered in cases where liver damage is probable or existing, and as an adjunct therapy during therapy for hepatocellular carcinoma, a prominent form of hepatic cancer [697,698,699,700,701,702,703,704].

Another interesting research avenue would be the comparative effectives of the different pinosylvin stereoisomers. For instance, trans-resveratrol has been found to have better anticancer properties compared to cis-resveratrol [705] and trans-stilbenes, in general, exhibit a better inhibitory action against COX [706].

A number of stilbene-based drugs, such as raloxifene and tamoxifen, are currently available [707]; in fact, the stilbene core is a very versatile structure with very diverse and promising potential uses [708]. Further development along this axis may prove successful and provide alternatives to existing solutions or new avenues for pharmacotherapy.

Regarding the phytochemical uses, there is a wealth of ethnomedical and ethnobotanical evidence on the uses of pinosylvin-producing plants; many of these uses are verified by recent research efforts. Apart from the medical traditions directly mentioned heretofore, there are various other local medical systems from ancient people, such as that of the Incas of Peru [709,710,711], the Aztecs of Central America [712,713], and the aborigines of Oceania [714,715]. In all these medical traditions, along with those already mentioned, from Asia, Europe, North America, and Africa, there are a host of foodstuffs used as medicines [716,717,718,719,720]; we propose that concerted research on the constituents of these foodstuffs be performed so that their stilbenoid content in particular be explored and relevant applications are experimentally tested.

**Figure 3:**
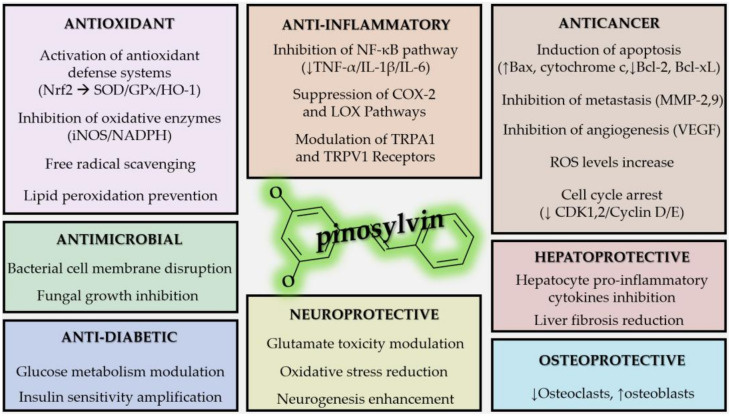
Summary of key biological activities and known action mechanisms involved. Nrf2 = nuclear factor E2-related factor 2; SOD = superoxide dismutase; GPx = glutathione peroxidase; HO-1 = heme-oxygenase 1; iNOS = inducible nitric oxide synthase; NADPH = nicotinamide adenine dinucleotide phosphate; NF-κB = nuclear factor kappa B; TNF = tumour necrosis factor; IL = interleukin; COX = cyclooxygenase; LOX = lipoxygenase; TRP = transient receptor potential; MMP = matrix metalloproteinase; VEGF = vascular endothelial growth factor; ROS = reactive oxygen species; CDK = cyclin-dependent kinase.

An overview of the most relevant and studied medical applications of pinosylvin and its derivatives is depicted in Figure 3.

### 11.2. Non-Medical Uses of Pinosylvin and Its Derivatives

It has also been reported that the bark of *P. roxburghii* has been tested for use in the removal of toxic waste from the water. Research results were encouraging at least in the case of Cr(VI) [579]. Pine bark has also been successfully tested in a similar capacity, with the addition of microalgae cultivated on it [721]; the use of pine bark instead of activated carbon is another possibility for pesticide removal [722]. Indeed, parts of plants have been explored as solutions in wastewater treatment and management systems in the last decades [723,724,725]. Heavy metal pollution is attributable both to mining in different sites and locations and industrial processes [650,726,727,728,729,730,731,732,733,734,735,736,737,738]; on the other hand, pesticide-associated pollution is an important issue both from historical and current uses [739,740,741,742,743]. Pesticides are a potential source of severe intoxication [744,745,746]. Currently, there are a number of initiatives and research regarding both the remediation of mining cites and the mitigation of pesticide pollution [747,748,749,750,751,752,753,754,755,756,757,758]. Perhaps, for such purposes, pinosylvin can be combined with or added to novel recovery mediums such as the ones proposed by a number of researchers [759,760,761].

It is important to mention at this point that parts of *P. halepensis* Mill possess a notable herbicidal activity [495,496,762,763,764]. Evidence suggests that the essential oils of the needles of this plant possess a larvicidal activity against the larvae of the Aedes albopictus mosquito [765]. On the other hand, up to a certain concentration, there were no discernible effects against the larvae of *Drosophila melanogaster* [493]. Moreover, *Stemona collinsiae* has also been used as an insecticide by the local people in Thailand—the ethanolic extract of the plant has been proven capable of eliminating the larval and adult stages of *P. ruficornis* [766] and the larvae of Musca domestica and *Chrysomya megacephala* [767].

A summary of the above-mentioned applications is available in Table 10.

These properties of *P. halepensis* Mill and *Stemona collinsiae* may prove important in the context of relevant policies of the European Union, which has developed and implemented a comprehensive policy and legal framework to regulate and promote the development and use of phytomedicines, plant-based substances, and natural products. First and foremost, it is widely known that the EU supports the use of plant-based substances as alternatives to synthetic chemical pesticides and herbicides, in an effort to reduce dependence on the latter [768,769]. Directive 2009/128/EC sets the ground rules for sustainable pesticide use, aiming to reduce health and environmental risks while promoting integrated pest management and alternative non-chemical methods [770]; the regulation (EC) No 1107/2009 lays down the main instruments for placing effective plant protection products (using pesticide substances) on the market that are safe for humans, animals, and the environment, while at the same time ensuring effective functioning of the internal market and improved agricultural production [771]. Meanwhile, the Horizon Europe framework [772] funds research into sustainable agriculture, including innovations in plant-based pesticides and herbicides. Therefore, it also aligns with the EU’s Farm to Fork strategy [773,774] under the European Green Deal [775] which aims to reduce pesticide and fertiliser use by 2030.

Moreover, there is phytoremediation which refers to the utilization of a plant-based substance for the removal or neutralization of contaminants like heavy metals in soil or wastewater [776,777,778]. The EU in the context of its broader sustainability goals, either by aligning with the United Nations (UN) 2030 Agenda which includes the 17 Sustainable Development Goals (SDGs) or by having sustainable development as its core principle (Art.3.3.) of the Treaty on European Union (TEU) [779,780] as well as implementing through strategies such as the European Green Deal, delineates and fosters phytoremediation directly and indirectly under the Waste Framework Directive (2008/98/EC) and the Water Framework Directive (2000/60/EC) [780,781,782]. Additionally, among the other EU policies and frameworks, there is the Urban Waste Water Treatment Directive (91/271/EEC) and its consolidated 2014 version [783,784,785] which promote the treatment of urban wastewater and encourage the exploration of innovative technologies, including plant-based and natural filtration methods.

Finally, the EU’s regulatory framework research and development programs have a significant focus on fostering innovation in plant-based substances offering at the same time funding opportunities through Horizon Europe, the largest research and innovation programme supporting projects in sustainable agriculture, biodiversity, and green technologies, as well as the LIFE Programme [772,786,787], the only EU funding initiative entirely dedicated to environmental, climate, and energy objectives and projects. Those initiatives and tools can easily support any aspiring phytoremediation and natural plant-based technologies/projects including but not limited to those with a pollution control orientation.

In the USA, biopesticides are regulated by the Environmental Protection Agency (EPA) under the Federal Insecticide, Fungicide, and Rodenticide Act (FIFRA) [788], which regulates specifically the distribution, sale, and use of pesticides while streamlining registration for biopesticides over chemical ones. Supplementarily, the Food Quality Protection Act (FQPA) is applicable and requires that all pesticides meet specific health standards, with an emphasis on sustainable use, while phytoremediation is guided by the National Environmental Policy Act | US EPA (NEPA), a US environmental law designed to promote the enhancement of the environment, and the Toxic Substances Control Act (TSCA) under EPA oversight [789,790,791].

In Japan, the Agricultural Chemicals Control Act [792] regulates all pesticides, including biopesticides, with a primary focus on safety. In the meantime, the Act on Promotion of Organic Agriculture [793] promotes sustainable use through reduced chemical inputs and supports integrated pest management (IPM) practices, in line with its environmental goals. Finally, the Environmental Conservation Law [794] enables phytoremediation projects, with a focus on urban and industrial applications under the oversight of the Japanese Ministry of the Environment.

In South Korea, the Framework Act on Environmental Policy promotes phytoremediation for soil health, aligning with its respective national environmental priorities which include but are not limited to sustainable development, low carbon, green growth, and environmental protection [795,796]. On another note, under the Agrochemicals Control Act [797], biopesticides must meet stringent safety standards. In addition, there is also the Sustainable Agriculture Promotion Act [798] that encourages reduced pesticide reliance, and supports organic farming initiatives, promoting IPM as part of sustainable agriculture practices.

Conclusively, each country’s regulatory framework is adjusted to unique national plans and policies, supporting overall reduced pesticide use and sustainable agricultural practices such as the introduction of safe biopesticides under certain conditions and fulfilling specific minimum requirements. Each country also seems to support phytoremediation either directly through funding or indirectly through its regulatory regime, yet we always need to consider the varying cultural and market priorities when comparing the state of play in multiple and diverse jurisdictions.

## 12. Conclusions

Pinosylvin and its derivatives represent a potent class of bioactive compounds which can be employed as antimicrobial and potentially as antiviral agents. At the same time, the antioxidant, anti-inflammatory, and anticancer actions of said compounds can be used to improve the actions of existing drugs and perhaps improve the prognoses in certain cases or reduce drug doses and associated side-effects. Questions of bioavailability can be addressed via the use of novel delivery systems. The multifaceted uses and applications of pinosylvin even comprise land remediation. Thus, it can be a potential contributor towards more environmentally friendly policies for insecticide and pesticide use, in accordance with global trends and European Union directives.

## Figures and Tables

**Table 1:** Taxonomical classification of pinosylvin-producing plants (listed alphabetically by species).

Order	Family	Genus	Species	Reference
Fabales	Fabaceae	*Arachis*	*A. hypogaea*	[51]
		*Cajanus*	*C. cajan*	[53]
Rosales	Rhamnaceae	*Hovenia*	*H. dulcis* Thunb.	[54]
Laurales	Lauraceae	*Lindera*	*L. reflexa* Hemsl.	[55]
Myrtales	Myrtaceae	*Agonis*	*A. flexuosa*	[56]
Pinales	Pinaceae	*Picea*	*P. abies*	[57]
			*P. glauca*	[58]
		*Pinus*	*P. banksiana*	[59,60,61]
			*P. brutia* Hen.	[62]
			*P. caribaea*	[63]
			*P. cembra*	[64]
			*P. contorta*	[64]
			*P. densiflora*	[65,66]
			*P. halepensis* Mill.	[67,68]
			*P. merkusii*	[69]
			*P. nigra* Arn.	[70]
			*P. palustris*	[71]
			*P. pinaster*	[72,73,74]
			*P. resinosa*	[60]
			*P. roxburghii* Sargent	[75]
			*P. strobus*	[71]
			*P. sibirica*	[64]
			*P. sylvestris*	[76]
			*P. taeda*	[77]
Pandanales	Stemonaceae	*Stemona*	*S.* cf. *peirrei*	[78]
			*S. collinsae*	[79,80]
			*S. tuberosa*	[81]

**Table 3:** Antibacterial actions of pinosylvin and its derivatives based on current research (reported alphabetically based on genera).

Genus	Species	Tested Substance	Extract Origin	Effectiveness	Year	Reference
Achromobacter	*A. xylosoxidans*	Pinosylvin, pinosylvin monomethyl ether	*P. banksiana*, *P. contorta*, *P. resinosa*, *P. sylvestris*	11–20 (and more) mm ^1^	2004	[60]
Arcobacter	*A. butzleri*	Pinosylvin	n/a (pure compound)	128 μg/mL (MIC)	2019	[139]
Bacillus	*B. cereus*	Pinosylvin, pinosylvin monomethyl ether, dihydropinosylvin monomethyl ether	*P. strobus*, *P. sylvestris*	101 ± 6, 92 ± 4, 82 ± 3 (% of inhibition)	2007	[140]
	*B. coagulans*	Pinosylvin, pinosylvin monomethyl ether	*P. contorta*, *P. banksiana*, *P. resinosa*, *P. sylvestris*	16–20 (and more) mm ^1^	2004	[60]
	*B. subtilis*	Pinosylvin	n/a (laboratory synthesis)	64 μg/mL (MIC)	2016	[141]
		Pinosylvin	n/a (pure compound)	99.2% ^2^	2019	[142]
Burkholderia	*B. multivorans*	Pinosylvin, pinosylvin monomethyl ether	*P. contorta*, *P. banksiana*, *P. resinosa*, *P. sylvestris*	11–20 (and more) mm ^1^	2004	[60]
Campylobacter	*C. coli*, *C. jejuni*	Pinosylvin	n/a (pure compound)	25–50 μg/mL (MIC)	2015	[143]
		Pinosylvin	n/a (pure compound)	Multiple values of inhibition haloes based on experimental parameters	2018	[144]
Escherichia	*E. coli*	Pinosylvin	n/a (pure compound)	250 μg/mL (MIC)	2005	[145]
		Pinosylvin, pinosylvin monomethyl ether, dihydropinosylvin monomethyl ether	*P. strobus*, *P. sylvestris*	54 ± 8, 71 ± 7, 18 ± 2 (% of inhibition)	2007	[140]
		Pinosylvin	n/a (laboratory synthesis)	64 μg/mL (MIC)	2016	[141]
		Pinosylvin	n/a (pure compound)	58.9% ^2^	2019	[142]
Listeria	*L. monocytogenes*	Pinosylvin, pinosylvin monomethyl ether, dihydropinosylvin monomethyl ether	*P. strobus*, *P. sylvestris*	62 ± 15, 100 ± 7, 64 ± 12 (% of inhibition)	2007	[140]
		Pinosylvin	n/a (laboratory synthesis)	93.2 ± 0.4 (% of inhibition at concentration of 0.5 mM)	2013	[146]
		Pinosylvin	n/a (pure compound)	97.9% ^2^	2019	[142]
Proteus	*P. vulgaris*	Pinosylvin	n/a (laboratory synthesis)	>128 μg/mL (MIC)	2016	[141]
Pseudomonas	*P. aeruginosa*	Pinosylvin	n/a (laboratory synthesis)	>128 μg/mL (MIC)	2016	[141]
		Pinosylvin	n/a (pure compound)	8.9% ^2^	2019	[142]
	*P. fluorescens*	Pinosylvin, pinosylvin monomethyl ether, dihydropinosylvin monomethyl ether	*P. strobus*, *P. sylvestris*	50 ± 15, 35 ± 2, 22 ± 6 (% of inhibition)	2007	[140]
Salmonella	*S. infantis*	Pinosylvin, pinosylvin monomethyl ether, dihydropinosylvin monomethyl ether	*P. strobus*, *P. sylvestris*	42 ± 20, 40 ± 7, 14 ± 2 (% of inhibition)	2007	[140]
	*S. enteritidis*	Pinosylvin	n/a (laboratory synthesis)	80.6% ^2^	2019	[142]
Staphylococcus	*S. aureus*	Pinosylvin	n/a (pure compound)	250 μg/mL (MIC)	2005	[145]
		Pinosylvin, pinosylvin monomethyl ether, dihydropinosylvin monomethyl ether	*P. strobus*, *P. sylvestris*	76 ± 2, 105 ± 12, 76 ± 4 (% of inhibition)	2007	[140]
		Pinosylvin	n/a (laboratory synthesis)	75 μg/mL MIC	2013	[146]
		Pinosylvin	n/a (laboratory synthesis)	64 μg/mL (MIC) (higher for some MRSA strains)	2016	[141]
		Pinosylvin	*A. hypogea*	≤100 μg/mL (MIC)	2018	[147]
		Pinosylvin	n/a (pure compound)	100% ^2^	2019	[142]
	*S. epidermidis*	Pinosylvin	n/a (laboratory synthesis)	128 μg/mL (MIC)	2016	[141]

^1^ inhibition zone diameter—different diameters depending on extract source; ^2^ bacterial load difference. MIC = minimum inhibitory concentration.

**Table 4:** Antifungal actions of pinosylvin and its derivatives based on current research (reported alphabetically based on genera).

Genus	Species	Tested Substance	Extract Origin	Effectiveness	Year	Reference
Aspergillus	*A. fumigatus*	Pinosylvin, pinosylvin monomethyl ether, dihydropinosylvin monomethyl ether	*P. strobus*, *P. sylvestris*	14 ± 1/ 12 ± 3 ^1^	2007	[140]
Candida	*C. albicans*	Pinosylvin	n/a (pure compound)	62.5 μg/mL (MIC)	2005	[145]
		Pinosylvin, pinosylvin monomethyl ether, dihydropinosylvin monomethyl ether	*P. strobus*, *P. sylvestris*	85 ± 5/ 80 ± 4 ^1^	2007	[140]
Cladosporium	*C. herbarum*	Pinosylvin, 4′-methylpinosylvin	*S. collinsae*	n/a (only structurally similar stilbenoids were tested)	2002	[79]
		Pinosylvin, dihydropinosylvin	*S.* cf. *pierrei*	10mg/mL (EC_50_)	2004	[78]
Plasmopara	*P. viticola*	Pinosylvin, pinosylvin monomethyl ether	*P. pinaster*	23, 18 μΜ (available as IC_50_)	2017	[73]
Penicillium	*P. brevicompactum*	Pinosylvin, pinosylvin monomethyl ether, dihydropinosylvin monomethyl ether	*P. strobus*, *P. sylvestris*	15 ± 1/ 14 ± 2 ^1^	2007	[140]
Saccharomyces	*S. cerevisiae*	Pinosylvin	n/a (pure compound)	125 μg/mL (MIC)	2005	[145]
		Pinosylvin, pinosylvin monomethyl ether, dihydropinosylvin monomethyl ether	*P. strobus*, *P. sylvestris*	82 ± 13/ 35 ± 21 ^1^	2007	[140]

^1^ percentage of inhibition zone compared to control.

**Table 7:** Experiments on the anticancer properties of pinosylvin and its derivatives.

Compound	Plant	Tested on	Mechanism	Effect	Concentration/ Administration	Year	Reference
Pinosylvin	n/a (laboratory synthesis)	In vitro—Raji and Molt human lymphoblastoid cell lines	Inhibition of protein uptake	Antiproliferative	Direct addition in culture—15, 30 μg/ml	1986	[339]
Pinosylvin	*P. sylvestris*	In vitro—MCF-7 or T-47D breast cancer cells	Increase in oestrogen expression	Pro-proliferative	Dilution in ethanol and culture for 7 days—1 pM to 1 μΜ	1996	[344]
Pinosylvin	*P. sylvestris*	In vitro—RAW264.7 cells and HT-9 human colon cancer cells	COX-2 inhibition, free radicals scavenging and xanthine oxidase activity inhibition	Antiproliferative	n/a	2003	[359]
Pinosylvin, pinosylvin monomethyl ether, pinosylvin dimethyl ether	*P. resinosa* Ait.	In vitro—lung carcinoma cells A549 lung carcinoma cells, DLD-1 human colorectal adenocarcinoma cells, WS1 healthy cells	Unknown	Cytotoxic	Incubation for 48 h—different concentrations (IC_50_ = ~41–130 μg/mL depending on cell line; value for extract)	2008	[356]
Pinosylvin	n/a (laboratory synthesis)	In vitro—HT1080 human fibrosarcoma cells	Decreased expression of matrix metalloproteinases	Antimetastatic	Treatment of cells at almost confluency, with pinosylvin—12.5, 25 and 50 μM	2012	[347]
		In vivo—mice	Downregulation of COX-2 expression, decreased ERK1/2 and Akt phosphorylation and reduced metalloproteinase expression		Intraperitoneal administration—10 mg/kg per body weight		
Pinosylvin	n/a (laboratory synthesis)	In vitro—HTC 116 human colorectal cancer cells	Src/ERK and GSK-3/β-catenin signalling suppression	Antiproliferative	Incubation for different periods—various concentrations (depending on different experimental protocols); IC_50_ = 48.2 μM (for 24 h)	2013	[340]
Pinosylvin methyl ether	n/a (pure compound)	In vitro—LNCaP prostate cancer cells, RWPE-1, and EP156T non-malignant cancer cells	Alteration of cell cycle-related genes, modification of steroid and cholesterol biosynthesis and androgen-signalling	Antiproliferative	Incubation over a span of 3 days (EC_50_ = 250 nM)	2016	[341]
Pinosylvin	n/a (pure compound)	In vitro—THP-1 and U937 human monocytic cell lines	Caspace-3 activation, flipflop of phosphatidylserine, p62 degradation and LC3-II accumulation, downregulation of AMPKa expression	Cytotoxic	Pretreatment with pinosylvin—0 to 100 μmol/L (IC_50_ = ~20–30 μmol/L)	2018	[357]
Pinosylvin	n/a (pure compound)	In vitro—SAS, SCC-9, and HSC-3 cells	Inhibition of MMP-2, upregulation of TIMP-2 expression, and downregulation of the ERK1/2 signalling pathway	Antiproliferative and anti-metastatic	Direct addition in culture medium—20, 40, 80 μΜ	2019	[263]
Pinosylvin	n/a (pure compound)	In vitro—NPC-039, NPC-BM and RPMI 2650 cells (nasopharyngeal carcinoma cells)	Inhibition of MMP-2, and decreased expression of MMP-2 and MMP-9; decrease in vimentin and N-cadherin and E-cadherin expression and of ZO-1	Antimetastatic	Treatment of cells with pinosylvin—20, 40, 80 μΜ	2021	[348]
Pinosylvin	n/a (pure compound)	In vitro—ECA109 and TE1 oesophageal cancer cells	Reduction in syntaxin-6 and integrin α3 expression and enhanced vasodilator-stimulated phosphoprotein expression	Antimetastatic	Treatment of cells with pinosylvin for 24 h—0, 10, 20, 40, and 80 μM	2023	[354]

**Table 8:** Experiments on the neuroprotective effects of pinosylvin and its derivatives.

Compound	Plant	Tested on	Mechanism	Effect	Concentration and Administration	Year	Reference
Pinosylvin	n/a (pure compound)	In vitro—OGD/R-damaged PC12 cells	Upregulation of PINK1/Parkin-regulated mitophagy, upregulation of the Nrf2 pathway	Neuroprotection	Pretreatment of cells with pinosylvin for 24 h—10 μΜ	2021	[364]
		In vivo—rats	Reduction in infarct size and of neural cell death	Decrease in brain function loss	Intraperitoneal injection—50 mg/kg		

**Table 9:** Traditional ethnobotanical uses of pinosylvin-producing plants and experimentally verified actions.

Plant		Traditional/ Ethnobotanical Uses	Medical System	Tested Actions	Region/ Populations	References
Tax. Name	Common Name					
*A. hypogaea*	Peanut	Treatment of sleep disorders, antidiabetic, antilipidemic, weight loss, anticancer use, cardiovascular pathologies and haemorrhages, treatment of bronchitis, antibacterial	Traditional Chinese medicine, Nigerian folk medicine	Treatment of sleep disorders, antimicrobial, antioxidant	China, Nigeria/Chinese, local population	[381,382,383]
*C. cajan*	Pigeon pea	Antidiabetic, stimulant, analgesic, treatment of haemorrhage, oral pathologies, laxative, lactation induction	Bangladesh medical tradition, Trinidad and Tobacco medical tradition, traditional Chinese medicine, Ayurveda	Antimicrobial, antidyslipidaemic, antidiabetic, antioxidant, anticancer, hepatoprotective	Bangladesh, Trinidad and Tobacco, China, India	[384,385,386,387]
*H. dulcis*	Japanese raisin tree	Alcohol detoxification (perhaps hepatoprotective)	Traditional Chinese medicine, traditional Japanese medicine, traditional Korean medicine	Alcohol detoxification, hepatoprotective effect, antioxidant, antidiabetic, antimicrobial, antilipidaemic, anti-allergic, anti-inflammatory, prokinetic	China, Japan, Korea/Chinese, Japanese, Koreans	[388,389]
*P. glauca*	White spruce	Antibacterial, treatment for sore mouth and strep throat, sinusitis, haemoptysis, wound treatment, chronic pain treatment	Carrier people’s medical practices, Canadian Boreal Forest people	Antimicrobial, organoprotective, antioxidant	Canada/Carrier people, indigenous populations	[390,391,392,393]
*P. brutia* Ten.	Turkish red pine	Antitussive, antidiabetic, treatment of gastrointestinal complains, tonic	Turkish folk medicine	Antifungal, antibacterial, (potential) treatment of acute lung injury, (potential) antioxidant action, anti-inflammatory	Turkey/local population	[394,395,396,397,398,399]
*P. densiflora* Sieb. et Zucc.	Korean red pine	Antihypertensive, anti-atherosclerotic, treatment of strokes, diabetes, cancer, and balding	Traditional Korean medicine	n/a	Korea/Korean people	[400]
*P. halepensis* Mill.	Aleppo pine	Respiratory pathologies, wound treatments, anti-inflammatory, urinary problems, GI ulcers, prostate infections, infertility, antiseptic, adrenal gland stimulant, antimicrobial, toothache, baldness	Local medical traditions	Antibacterial, antifungal, antioxidant, cytoprotective, anticancer (cytotoxic), anti-coagulant, anti-haemolytic, anti-inflammatory	Italy, Spain, Algeria, Morocco/Berber people, local populations	[68]
*P. nigra*	Black pine	Colds, cough, treatment of respiratory pathologies, furuncles, warts, treatment of teeth decay, digestive complaints	Local medical traditions and Austro-Hungarian pharmacopoeia	Antimicrobial, antioxidant (weak), food supplement, antiproliferative	Romania/local people of Transylvania	[401,402,403,404]
*P. mugo* Tura	Dwarf mountain pine, scrub mountain pine, Swiss mountain pine	Expectorant	Local medical traditions	n/a	Italy	[405,406]
*P. roxburghii* Sargent	Chir pine, long-leaved pine	Antiseptic, diarrhetic, diaphoretic, tonic, vermifuge (anthelmintic), rubefacient, spasmolytic, antioxidant, anti-inflammatory, treatment for ocular and ear pathologies, ulcer treatment, bronchitis treatment, treatment of skin diseases, treatment of blood diseases, treatment of snake and scorpion bites	Ayurveda, various local medical traditions	Antibacterial, antioxidant, anticonvulsant, anti-asthmatic, analgesic activity	Himalayas, Hindu Kush/local tribes	[407,408,409,410]
*P. sylvestris*	Scots pine, Baltic pine, European red pine	Treatment of asthma, cough and respiratory complaints, treatment of rheumatisms and varicose veins	Local medical traditions and Austro-Hungarian pharmacopoeia	Antioxidant, anti-inflammatory, food supplement	Romania/local people of Transylvania	[403,404,411]
*P. virginiana* Miller	Virginia pine	Treatment of skin ulcers and sores, baths for painful joints, and treatment of cold and fever	Native American traditional medicine	n/a	Cherokee Native Americans	[412]

**Table 10:** Non-medical applications of the properties of pinosylvin-containing plants.

Plant Part Used	Purpose/Activity	Mechanism	Year	Reference
Bark of *P. roxburghii*	Water purification	Removal of Cr(VI) via adsorption	2005	[579]
Pine bark		Organochlorine pesticides removal	2011	[722]
Pine bark acting as a substrate for biofilm formation		Phycoremediation	2020	[721]
Essential oils of different *Pinus* spp.	Insecticidal activity	Repellent and larvicidal activity against *A. albopticus* mosquito larvae	2015	[765]
Ethanolic extract from *S. collinsiae* roots		Elimination of *P. ruficornis* in larval and adult stages	2017	[766]
Ethanolic extract from *S. collinsiae* roots		Elimination of *C. megacephala* flies at the larval stage	2023	[767]

Cr(VI) = hexavalent chromium.

## Data Availability

No new data were created or analyzed in this study. Data sharing is not applicable to this article.
